# 2D and 3D Self‐Assembly of Fluorine‐Free Pillar‐[5]‐Arenes and Perfluorinated Diacids at All‐Aqueous Interfaces

**DOI:** 10.1002/advs.202401807

**Published:** 2024-05-24

**Authors:** Lawrence W. Honaker, Tu‐Nan Gao, Kelsey R. de Graaf, Tessa V.M. Bogaardt, Pim Vink, Tobias Stürzer, Gabriele Kociok‐Köhn, Han Zuilhof, Fedor M. Miloserdov, Siddharth Deshpande

**Affiliations:** ^1^ Laboratory of Physical Chemistry and Soft Matter Wageningen University & Research Wageningen 6708 WE The Netherlands; ^2^ Laboratory of Organic Chemistry Wageningen University & Research Wageningen 6708 WE The Netherlands; ^3^ Biobased Chemistry and Technology Wageningen University & Research Wageningen 6708 WG The Netherlands; ^4^ Bruker AXS GmbH 76187 Karlsruhe Germany; ^5^ Core Research Facility University of Bath Bath BA2 7AY UK; ^6^ School of Pharmaceutical Science and Technology Tianjin University Tianjin 300072 P. R. China; ^7^ China–Australia Institute for Advanced Materials and Manufacturing Jiaxing University Jiaxing 314001 P. R. China

**Keywords:** aqueous two‐phase systems, electrospray, fluorophilic aggregation, microfluidics, PFAS, pillar‐[5]‐arenes, supramolecular assembly

## Abstract

The interaction of perfluorinated molecules, also known as “forever chemicals” due to their pervasiveness, with their environment remains an important yet poorly understood topic. In this work, the self‐assembly of perfluorinated molecules with multivalent hosts, pillar‐[5]‐arenes, is investigated. It is found that perfluoroalkyl diacids and pillar‐[5]‐arenes rapidly and strongly complex with each other at aqueous interfaces, forming solid interfacially templated films. Their complexation is shown to be driven primarily by fluorophilic aggregation and assisted by electrostatic interactions, as supported by the crystal structure of the complexes, and leads to the formation of quasi‐2D phase‐separated films. This self‐assembly process can be further manipulated using aqueous two‐phase system microdroplets, enabling the controlled formation of 3D micro‐scaffolds.

## Graphical abstract

A class of cyclic compounds, pillar‐[5]‐arenes, can rapidly form unusual complexes with perfluorinated molecules, and this formation is facilitated by size matching between the two. Through microfluidics and aqueous two‐phase systems, these can be shaped into shape‐retaining vesicles at water–water interfaces.

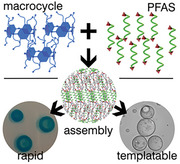

## 1 Introduction

Per‐ and polyfluoroalkyl substances (PFAS), widely used since the 1950's,^[^ ^1^ ^]^ have a unique set of physical properties, including hydrophobicity, lipophobicity, and chemical inertness, which makes them highly useful as components in new materials with unconventional physical and chemical properties.^[^ ^2^ ^]^ The nonstick properties of PFAS‐based materials particularly stand out, but their nonflammability and abrasion resistance have also contributed to their extremely widespread use. The unique combination of properties continues to lead to novel uses in a very wide range of fields. For instance, perfluorinated diacid (PFDA) can substantially decrease the influence of humidity on the conductivity of polyaniline^[^ ^3^ ^]^ and also enhances the loading capacity of perfluoro‐15‐crown‐5‐ether on covalent organic polymers, facilitating their subsequent application in the photodynamic treatment of tumors.^[^ ^4^ ^]^ The self‐assembly of fluorinated organic molecules has been further demonstrated for potential applications in drug delivery, energy storage, photosensitizers, and ^19^F nuclear magnetic resonance imaging.^[^ ^5^ ^]^ For these applications, fluorinated nanoparticles are commonly generated from block copolymers containing one or more fluorinated blocks.^[^ ^6^ ^]^ The varying degree of hydrophobicity among these blocks allows the polymer chain to fold and assemble into different morphologies depending on the solvent used. Nonetheless, notwithstanding the thus evident advantages of controlled use, it is clear that the ubiquitous presence of PFAS (which have, e.g., been detected on both poles^[^ ^7^ ^]^ and in the blood of newborn infants^[^ ^8^ ^]^) and the increasing evidence for the significant toxicological effects thereof demand those novel ways are to be used to bring the genie back into the bottle.

**Figure 1:**
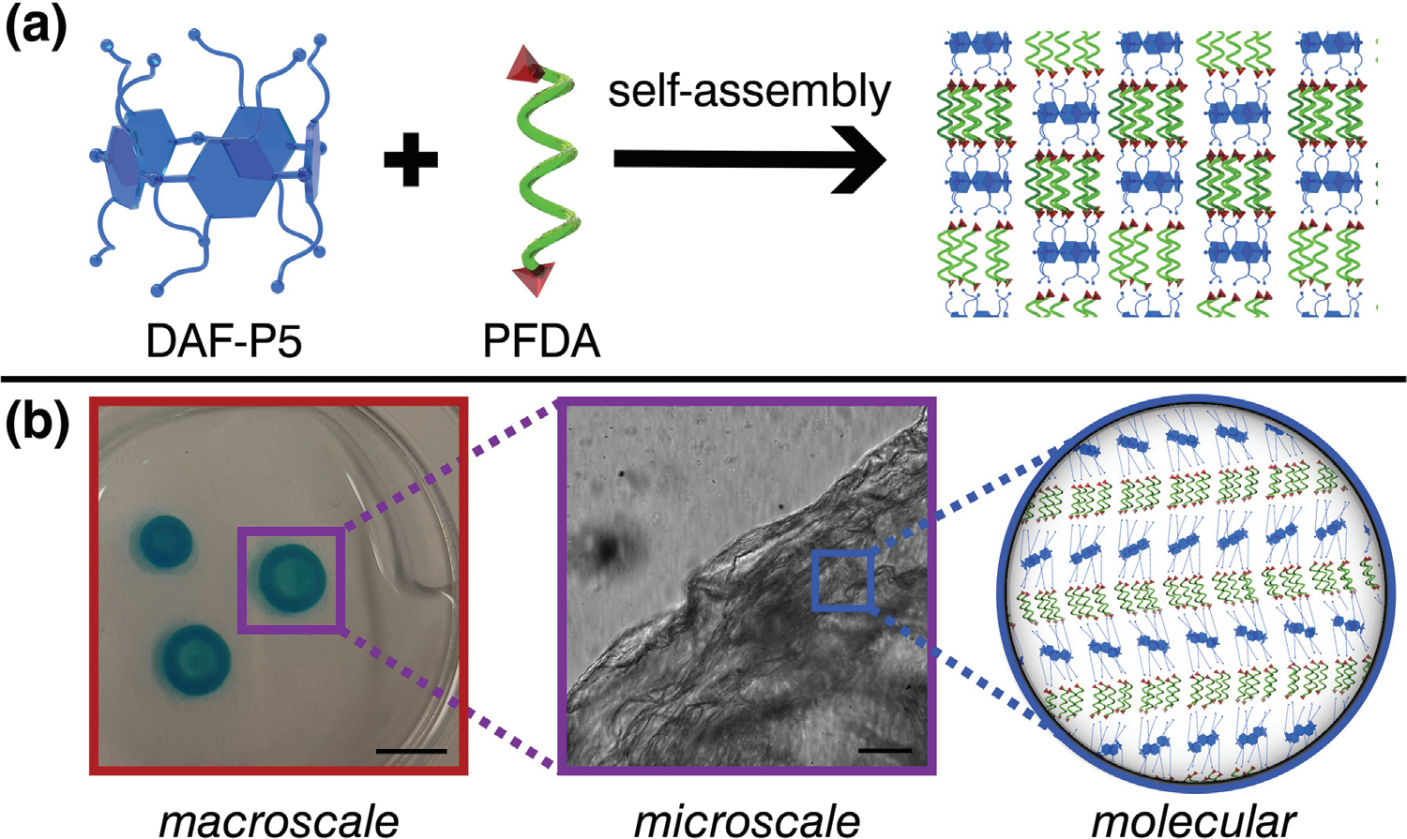
Pillar‐[5]‐arenes and perfluorinated diacids readily self‐assemble in an all‐aqueous environment to form macroscopic films. a) Schematic self‐assembly of DAF‐P5s with PFDA molecules. b) The assembly manifests itself at several scales: millimeter‐scale stable structures (food dye added for better visualization) arising from microscale assembly of quasi‐2D films, itself a consequence of molecular level interactions between the two components. Scale bar for macroscale 5 mm; for microscale 100 µm.

While the self‐assembly of organic polymers with PFAS and PFAS‐derived polymers is relatively well established, and while previous studies have reported the complexation between nonfluorinated and fluorinated molecules,^[^ ^9^ ^]^ we are not aware of significant examples of supramolecular self‐assembly between fluorinated and non‐fluorinated small molecules (*M_w _* ≤ 1 kDa) leading to the formation of macroscopic supramolecular structures. Recently, we discovered a new host–guest interaction between negatively charged PFAS, such as perfluorooctanoic acid (PFOA), perfluorooctanoic sulfonate (PFOS), and decaammonium functionalized pillar‐[5]‐arenes (DAF‐P5s).^[^ ^10^ ^]^ The unique structure of DAF‐P5s, where five positively charged pendant groups are positioned in a confined space with the same orientation,^[^ ^11^ ^]^ allows PFAS molecules to aggregate at the rims of the DAF‐P5s, creating a supramolecular complex (**Figure** **1**a). This behavior provides us with a new approach for inducing self‐assembly between unfluorinated and fluorinated small molecules.

Driven by these findings on mono‐acids, in this work, we report and characterize the rapid self‐assembly between perfluoroalkyl diacids (PFDA) and DAF‐P5 molecules and the subsequent formation of macroscopic supramolecular structures at all‐aqueous interfaces (Figure 1b). We find that the formation of structures is facilitated by fluorophilic antisolvent interactions and size‐matching effects, where the matching dimensions of the rigid components of both the P5 and the PFDA stabilize the formed assemblies. We also demonstrate the ability to shape the formed supramolecular structures through the use of aqueous two‐phase systems produced using microfluidics and electrospray techniques. These all‐aqueous microdroplets template the interfacial self‐assembly and enable the creation of vesicles with a rigid shell, a concept that could be potentially used for the controlled removal of PFAS from aqueous systems.

## 2 Results and Discussion

### 2.1 Pillar‐[5]‐Arenes and Perfluorinated Diacids Spontaneously and Rapidly Form Assemblies at Contacting Interfaces

We first performed bulk assays where 5 mm pillar‐[5]‐arene (P5) solution was added dropwise into an aqueous 5 mm perfluorosebacic acid (PFDA‐10) solution. Upon doing so, we observed the formation of a “film” at the contact area of the two solutions almost instantaneously (see Video S1 , Supporting Information). The formed scaffolds were macroscopically visible, maintaining the shape templated by the contact area, on the scale of several millimeters to centimeters (as seen in Figure 1b).

#### 2.1.1 Multivalency and Fluorination are Both Necessary to Drive Assembly

**Figure 2:**
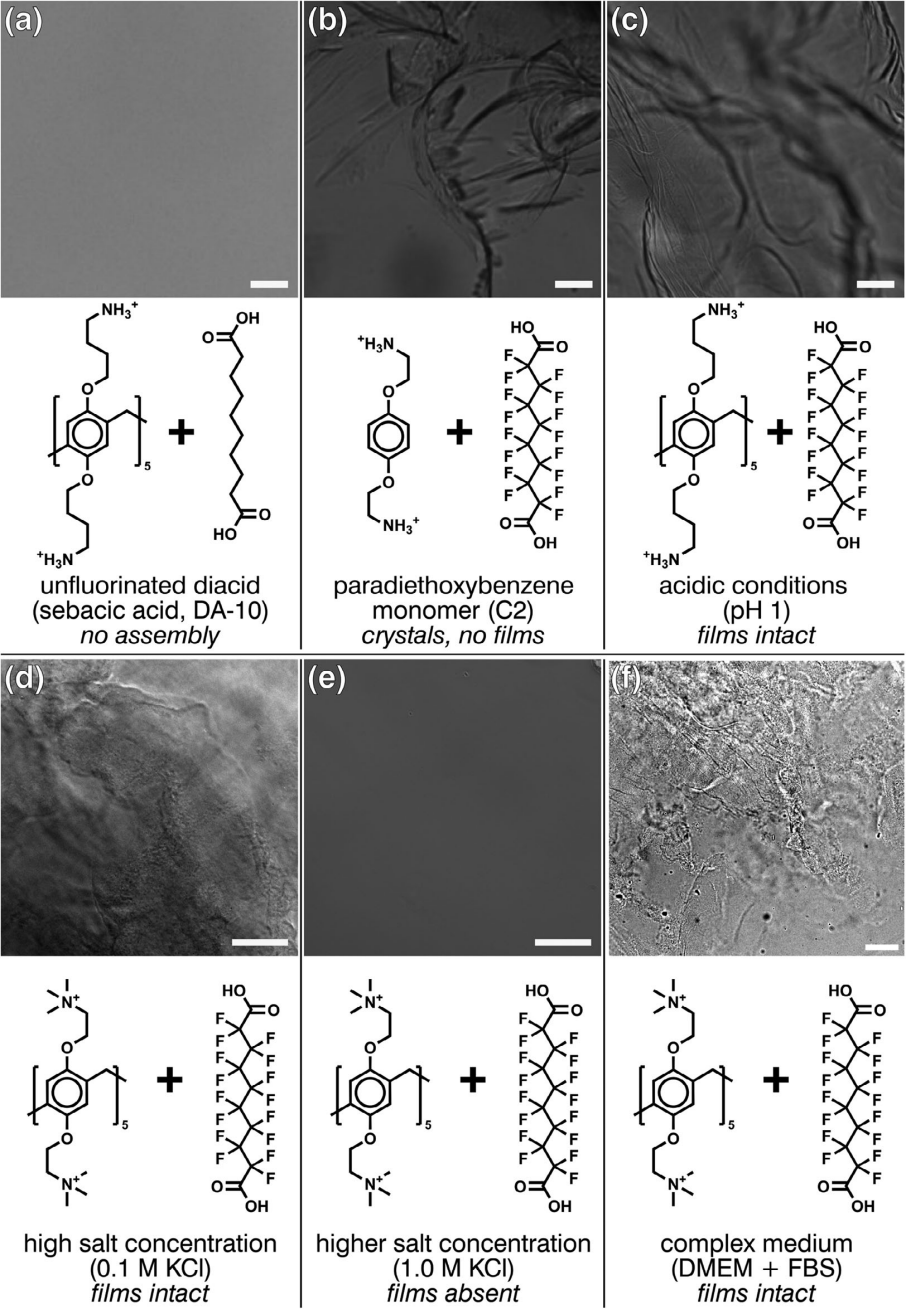
Perfluorination of the diacid and the preorganization provided by cyclization of the arene monomers are essential to the self‐assembly, while electrostatic screening effects are not as pronounced. a) Replacing the perfluorinated diacid with its unperfluorinated analogue (sebacic acid) results in no assembly. b) Replacing the pillar‐[5]‐arene with an arene monomer (*para*‐diaminoethloxybenzene) does produce crystals in a complex with perfluorosebacic acid, but these do not create a self‐assembled film. c) Changing the pH from 7.4 (the buffer pH) to a strongly acidic condition (pH 1) does not inhibit or strongly disrupt the self‐assembly. d,e) Moderate salt concentrations, such as 0.1 m KCl (aq) d), do not disrupt assembly either, with disruption only observed at 1.0 m KCl e). f) The use of a complex medium such as Dulbecco's Modified Eagle Medium (DMEM) with added fetal bovine serum (FBS) does not inhibit the formation of the structures, alluding to the robustness of the assembly process. Scale bars 100 µm. Images adjusted to improve contrast.

Upon observing the unique self‐assembly behavior of P5 with PFDA, we were prompted to investigate what molecular interactions allow perfluorinated molecules—traditionally considered to be very inert— to self‐assemble with P5. As shown in **Figure** **2**, we probed the molecular interactions by studying the possible complex formation with a systematically varied set of molecular analogs of PFDA‐10 and P5s and by altering the conditions under which the molecules interacted, which were all found to have different degrees of impact on the assembly process.

Using the assembly of P5C4 and PFDA‐10 as a baseline, we investigated the effects of fluorination and multivalency. First, we replaced PFDA‐10 with its unperfluorinated analog, sebacic acid (Figure 2a), finding that no assembly occurred, strongly hinting that the perfluorination of the diacid is a key aspect of the assembly. Furthermore, when we replaced the P5C4 pentamer with its analogous “monomer” (*para*‐diaminobutyloxybenzene) of the P5, as seen in Figure 2b, a crystalline precipitate was formed, but no clear film when using the “pentamer” (P5). This suggests that the presence of five charged groups in a confined space is crucial to the self‐assembly of the film.

As the self‐assembly occurs between DAF‐P5s that are expected to be polycationic upon dissolution in water and PFDAs, expected to be anionic upon dissolution in water, one would intuitively assume the self‐assembly reaction to be driven by a combination of charge–charge interaction and hydrogen bridges. We note that assemblies primarily driven by electrostatic interactions have been previously reported,^[^ ^12^ ^]^ but we wished to know whether these electrostatic interactions were the primary drivers of the assembly or if there were instead other factors driving the assembly process. Our hypothesis was probed by screening the charges of the molecules during self‐assembly with either low pH, high pH, or high ionic strength. A low pH (1.0; Figure 2c) or high pH (10) did not have any effect on the self‐assembly of the material, indicating that the self‐assembly is reliant on interactions that are not sensitive to pH within this wide range, though we note that the extremely low pKa of perfluorinated diacids^[^ ^13^ ^]^ renders them generally impervious to small changes of pH. Using P5C2M and PFDA‐10, we observed that the self‐assembly was unaffected at low ionic strength (0.1 m KCl, Figure 2d) but became disrupted at much higher ionic strength (1.0 m KCl, Figure 2e). Other combinations of P5 and PFDA showed a different degree of sensitivity to salt screening: for example, we observed disruption of film formation between P5C2 and PFDA‐10 even at 100 mm KCl, possibly owing to the nonpermanent charge of the terminal amine group on P5C2 that can be more readily screened compared to the trimethylated amine group of P5C2M. This suggests some reliance on charge–charge interactions between the anionic PFDAs and the cationic P5s, but we can conclude that, in general, the self‐assembly reaction is not as strongly driven by charge–charge interactions or the formation of hydrogen bonds as it is by the fluorophilic interactions.

To further probe the binding mechanism, we also attempted to perform the self‐assembly in a complex biological environment. We chose Dulbecco's Modified Eagle Medium (DMEM), commonly used to grow cell cultures, as an environment in which to grow the films, both with and without added fetal bovine serum (FBS). We found that the presence of such a complex environment did not negatively affect the self‐assembly, as seen in Figure 2f, showing the extreme robustness and bio‐orthogonal nature of the underlying process. It further suggests that perfluorinated diacids are capable of self‐assembling even in complex biological systems such as extracellular fluid, further underlining the role of fluorophilic aggregation in the self‐assembly process. Finally, we found that the relative concentrations of the PFDA and the P5 did affect the assembly process (as seen in Figure S10, Supporting Information). However, this effect was limited to the observation of threshold concentrations and ratios at or above which assembly would occur rather than affecting the morphology of the films. Above 0.63 mm PFDA‐10 and 0.25 mm P5C4, films were consistently observed, regardless of the concentration of either the P5 or PFDA, while hints of assembly (albeit not in film form) could be observed with other concentration pairs. Notably, if the concentration was below the threshold, we could not derive a binding constant with ITC (Figure S8a, Supporting Information), while the stoichiometric binding ratio was found to be independent of concentration above the threshold (Figure S8b, Supporting Information).

#### 2.1.2 The P5–PFDA Assembly is Driven by Fluorophilic Aggregation

With the two‐component assembly readily generated and interactions beginning to be understood, we sought to improve our comprehension of the forces driving the self‐assembly. To do so, we synthesized a variety of symmetric P5s using established protocols,^[^ ^14^ ^]^ varying the length of the pendant alkyl tails of DAF‐P5s from two to six carbons (P5C2, P5C4, and P5C6). In addition, a two‐carbon variant was made with a trimethylated amine terminal group (P5C2M), which thus displays a permanent positive charge, but with no possibility to form hydrogen bridges. We also used four different PFDA variants (from six to twelve carbons; PFDA‐6, PFDA‐8, PFDA‐10, and PFDA‐12). This set of compounds allowed us to systematically investigate the effect of chain length on the supramolecular assembly between P5s and diacids.

**Figure 3:**
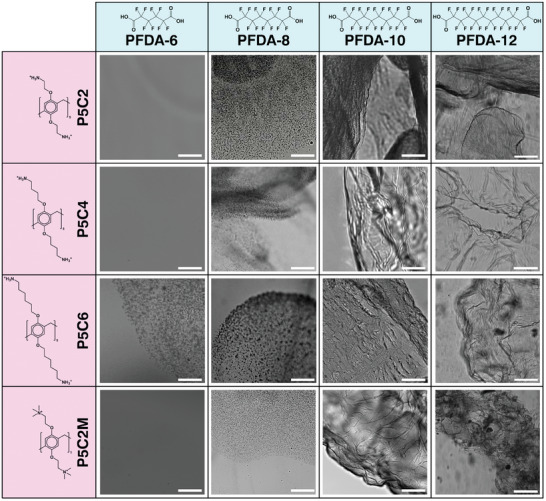
Structure formation is dependent on the relationship between the pendants of the pillar‐[5]‐arene and the lengths of the perfluorinated diacids. By maintaining a constant molar concentration of P5 (8 mm) and diacid (17 mm PFDA‐6, PFDA‐8, and PFDA‐10; ≈9 mm PFDA‐12), we could characterize three distinct regimes: no assembly (homogeneous solutions), weak assembly (grainy precipitates), and robust assembly (film formation). Film formation was only achievable by using perfluorinated sebacic acid (PFDA‐10) or perfluorinated dodecanoic acid (PFDA‐12). The use of PFDA‐12 resulted in visible films, but with greater fragility than when using PFDA‐10. The best structure formation was found between either P5C4 or P5C2M and PFDA‐10. In general, the identity of the P5 molecule mattered considerably less than the identity of the diacid. These data suggest that a size‐matching effect between the core of the P5 and the length of the rigid PFDA is necessary to facilitate the formation of films: the pendants of the P5 can accommodate the tethering of longer PFDA molecules, but they cannot accommodate shorter ones, causing film formation to fail with PFDA‐6 and PFDA‐8. Scale bar 300 µm.

We thus obtained an overview of the film‐forming capabilities of all combinations of P5 molecules and diacids, as shown in **Figure** **3**. Remarkably, when using perfluorinated sebacic acid (PFDA‐10), we obtained films with each of the four P5 variants we investigated, but we did not see a similar assembly with any combinations when a shorter perfluorinated diacid was used (either adipic acid, PFDA‐6, or suberic acid, PFDA‐8). We did obtain rapid, almost instantaneous film formation when using perfluorinated dodecanoic diacid (PFDA‐12, see Video S1, Supporting Information), but the formed films were more fragile (were disrupted easily upon gentle shaking) compared to when PFDA‐10 was instead used. We believe this to be a consequence of the length of the rigid, fluorinated backbone of the diacid “matching” to the size of the P5 molecules that facilitate the formation of lateral hydrogen bridges. The character of the pendant groups of the P5 influences the assembly to a moderate extent by affecting the physical properties of the final structures. For example, film formation with P5C4 appeared to be much less fragile compared to film formation with P5C2. Nonetheless, the role of the P5 tail length appears to be much less important compared to the role of the P5 core (as was observed in Figure 2b).

We further probed the interaction between P5C4 and PFDA with isothermal titration calorimetry (ITC) to better elucidate the molecular structure of the complexes. PFDA‐6 and PFDA‐8 both showed binding constants lower than 10^3^ m ^−1^ with P5C4, values below the accurate detection for ITC. PFDA‐10 shows a binding constant of (4.52 ± 3.01) × 10^4^ m ^−1^, with the binding ratio between P5C4 and PFDA‐10 determined to be 1:5 (see Table S2 and Figure S7, Supporting Information). This ratio was additionally confirmed by ^1^H and ^19^F NMR spectroscopy with an internal standard (see Figures S5 and S6, Supporting Information). Sebacic acid, the non‐fluorinated counterpart of PFDA‐10, was found to have a binding constant of (3.53 ± 0.17) × 10^6^ m ^−1^ and a stoichiometric binding ratio of 1:1. This difference in the binding ratio is indicative of a fundamentally different assembly behavior: unfluorinated sebacic acid will preferentially localize inside the P5 cavity, a classic example of a host–guest interaction, whereas its perfluorinated analog will assemble at the rims, stabilized by fluorophilic phase separation. By further elongating the PFDA to twelve carbons (perfluorododecanoic diacid), we found P5C4 with PFDA‐12 assembles in a 1:5 ratio with a very high binding constant, (7.61 ± 2.56) × 10^6^ m ^−1^, but this increased binding constant does not appear to be reflected in improved film resilience: the films obtained from assembly between PFDA‐12 and P5C2, P5C4, or P5C6 all rapidly disintegrated upon even gentle stresses (such as shaking the Petri dish in which they were formed), and the films from P5C2M and PFDA‐12 in bulk readily disintegrated even without any agitation.

ITC data additionally confirmed that, in the complex between P5C2 and PFDA‐10, salt could disrupt the formation of complexes, as seen in Figure S8c,d, Supporting Information).

These data, both spectroscopic and imaging, have several implications. First, they suggest the formation of P5–PFDA complexes, in which each of the P5 molecules is “crowned” by five PFDA molecules that are tethered to the P5 by the charged pendant groups. These PFDA units can then tether the next P5, creating an overall linear assembly and yielding a material consisting of supramolecular polymers. Second, the perfluorinated molecules need to have a minimal perfluorinated length in order to generate films. The formation of salt bridges alone is not sufficient to drive film formation with P5; the additional drive of fluorophilic antisolvent interactions is required. We notice that a grainy precipitate is visible with the combinations of P5 and PFDA‐8, a hint of the beginning of self‐assembly, but stable films only begin to emerge with PFDA‐10. Third, the stability of the assembled film is not solely determined by the linear interaction of P5 and PFDA, which is described by their binding constant. The greatest binding constants are observed when using PFDA‐12, yet the most robust self‐assemblies are formed between P5C4 and PDFA‐10. This suggests that, while binding constants do play a role, size‐matching effects are also crucial in stabilizing the assembled structures.

### 2.2 The Crystal Structure Shows the Formation of Unusual, Rod‐Shaped Assemblies

**Figure 4:**
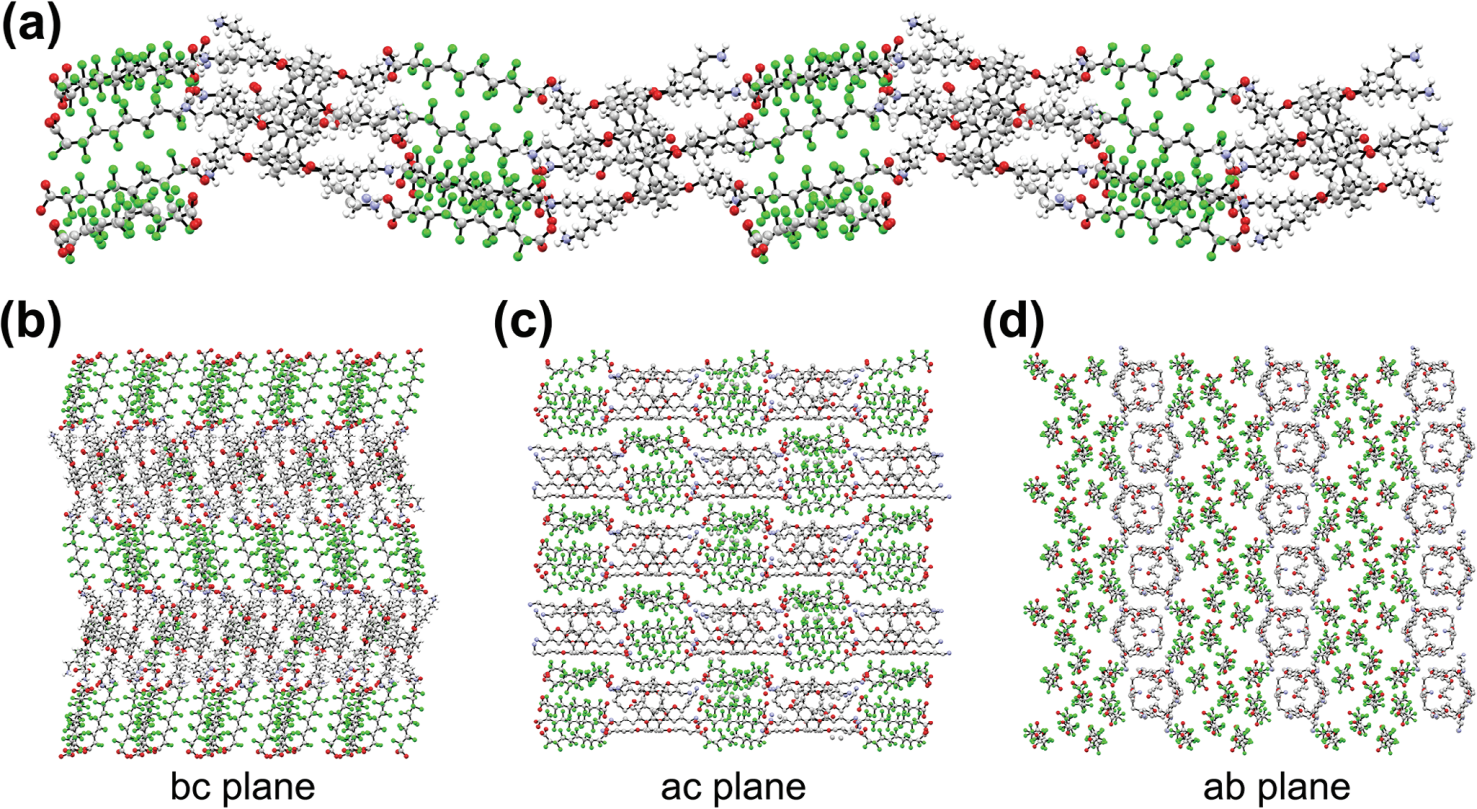
Single‐crystal X‐ray diffraction unveils the 3D structure of the P5–PFDA complex. a) The overall structure shows a linear stacked zig‐zagging arrangement of the PFDA‐10–P5C4 complex, with alternating layers of PFDA‐10 and P5C4. b) In the *bc* plane, a smectic‐like ordering is present, with the PFDA‐10 and P5C4 showing alternating layers. c) The *ac* plane, however, shows a “checkerboard” structure involving shoulder‐to‐shoulder stacking of alternating domains of PFDA‐10 and P5C4. d) The *ab* plane confirms the smectic‐like ordering observed in the *bc* plane.

To further elucidate detailed information about the structure of the self‐assembled complex, we grew P5C4–PFDA‐10 crystals in methanol and performed single crystal X‐ray diffraction in order to determine the spatial arrangement and orientation of the components of the P5C4–PFDA‐10 complex. As shown in **Figure** **4**, P5C4 and PFDA‐10 were found to form a linear zig‐zagging structure. Five PFDA molecules each are joined between two adjacent P5C4 rims, an arrangement in agreement with the stoichiometric ratio we obtained from both ITC and NMR spectroscopy. In the *bc* and *ab* crystallographic planes, the individual P5–PFDA lines are stacked “shoulder‐to‐shoulder” in a smectic‐like structure, the linear fluorous phase clearly distinct and observable in Figure 4b,d. However, in the *ac* plane (Figure 4c), we observed a staggered, checkerboard pattern, which means the fluorous phase is not an entirely lamellar structure. The staggered alignment in the ac plane might be caused by a size‐match effect: the fluorinated backbone of the diacid is almost the same length as the P5 *para*‐dioxybenzene core, which enables the formation of a phase‐separated, rigid, staggered structure rather than a lamellar‐like layered structure. This can be confirmed in part by the fact that we obtain visible self‐assembly with PFDA‐10 and any P5 host, regardless of the length of the alkyl tail (the tail length only moderately affects the quality of the self‐assembled structure), but we fail to obtain good structures with the smaller PFDA molecules, and we get much weaker films (more prone to breakage) with a longer PFDA. This checkerboarding, facilitated by size matching between the rigid PFDA and the aromatic core of the P5, can thus prevent interlayer shear gliding, which further stabilizes the structure of both the crystal and potentially that of the self‐assembled film. The additional fragility of the films formed with longer PFDA molecules may be due to the rigidity of the complexes being compromised by the additional energy requirement of stretching the P5 alkyl pendants to accommodate forming assemblies with the longer PFDA.

### 2.3 3D Assembly of P5–PFDA Can be Templated by Using Aqueous Two‐Phase Systems

After characterizing the P5‐PFDA self‐assembly at the water–water interface, we wondered if we could further direct and regulate the process in a 3D fashion and at a microscopic scale. To achieve this, we employed microfluidic and electrospray techniques in combination with an aqueous two‐phase system (ATPS) consisting of solutions of the polymers poly(ethylene glycol) (PEG) and dextran (DEX), a well‐established ATPS model system.^[^ ^15^ ^]^ We dissolved PFDA‐10 (17 mm) in the PEG solution and a P5, normally P5C2M (10 mm), in the DEX solution. The self‐assembly was compatible with ATPS components, confirmed by a rapid film formation at the interface when the two solutions were loaded on top of each other (Figure S11, Supporting Information).

**Figure 5:**
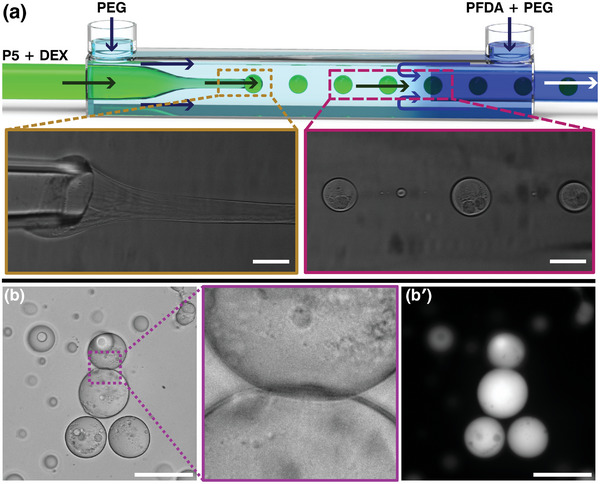
An aqueous two‐phase system, combined with microfluidics, can be used to template the assembly of pillar‐[5]‐arenes and perfluorinated diacids. a) A schematic of a glass‐capillary flow‐focusing microfluidics device that can create phase‐separated dextran droplets containing the P5, and the subsequent addition of PFDA to stable droplets then yields vesicle‐like structures at the droplet interface. b) After the droplet production and addition of perfluorinated diacid, the droplets do not show coalescence and remain separated even when in contact with each other, and (bʹ) corresponding epifluorescence micrographs show that fluorescently tagged dextran remains sequestered within the shell. Scale bars in (a) 100 µm; in (b) and (bʹ) 200 µm.

We then sought to replicate the macroscale assembly at the microscale and utilized ATPS droplets as a template to form closed 3D DAF‐P5–PFDA structures. To do so, we constructed glass capillary flow‐focusing microfluidic devices, commonly used to produce droplets and shells of immiscible fluids.^[^ ^16^ ^]^ Consisting of two inlets and one outlet, we operated such a device using a pressure‐driven flow to create droplets of P5C2M‐containing DEX solution (with a small amount of fluorescein isothiocyanate‐tagged DEX, FITC‐DEX, for epifluorescence visualization) in the PFDA‐containing continuous PEG phase. However, this led to very rapid self‐assembly at the capillary orifice and clogging of the device before successful ATPS droplet formation could be achieved. We mitigated this by adapting the microfluidic set‐up to have three inlets and one outlet in order to first create P5‐laden DEX droplets in a diacid‐free PEG solution before introducing them to a PFDA‐containing PEG outer phase, as depicted in **Figure** **5**a. Additionally, since the interfacial tension between the two systems is extremely low (on the order of µN m^−1^)^[^ ^15a,b^ ^]^ and the viscosity of the two solutions too high to rely on droplet generation through the development of Plateau–Rayleigh instability alone,^[^ ^17^ ^]^ we pulsed the flow rate of the DEX solutions to stimulate the breaking of the jet and consequently form DEX droplets in the PEG phase. In this way, we were able to obtain DEX droplets in glass capillary devices with a narrow size distribution (57.4 ± 3.0 µm, mean ± standard error, *n > *170). These adjustments not only allowed the ATPS droplets to be cleanly and stably produced, but they were also sufficient to initiate the P5–PFDA assembly at the droplet–water interface. The droplets collected at the device outlet did not coalesce, suggesting P5–PFDA self‐assembly occurred at the droplet boundaries, as seen in Figure 5b.

These droplets additionally showed an irregular/aspherical boundary instead of a smooth one, indicative of the development of a P5–PFDA film at the interface.^[^ ^18^ ^]^ Epifluorescence microscopy (Figure 5b′) confirmed that FITC‐DEX remains sequestered within the vesicle‐like structures.

These glass capillary experiments made us realize that some degree of flow of the PEG‐PFDA solution over the P5 droplets was essential to recruiting enough PFDA to the interface to form stable films: the DEX and PEG solutions are both viscous, leading to low Péclet numbers and thus hampering the diffusion of free PFDA molecules to the surface of the droplet. This led us to explore a more suitable production approach in the form of electrospray. Electrospray ionization is a technique where a potential difference is used to elongate a droplet (a “Taylor cone”^[^ ^19^ ^]^) into a jet and eventually break the jet into droplets.^[^ ^20^ ^]^ Electrospray has been previously used to generate interfacial assemblies.^[^ ^20c^ ^]^ Moreover, because electrospray operates by actively breaking the jet as opposed to the passive breaking achieved with Plateau–Rayleigh instability, one would, in principle, expect to generate a more uniform droplet size distribution.

**Figure 6:**
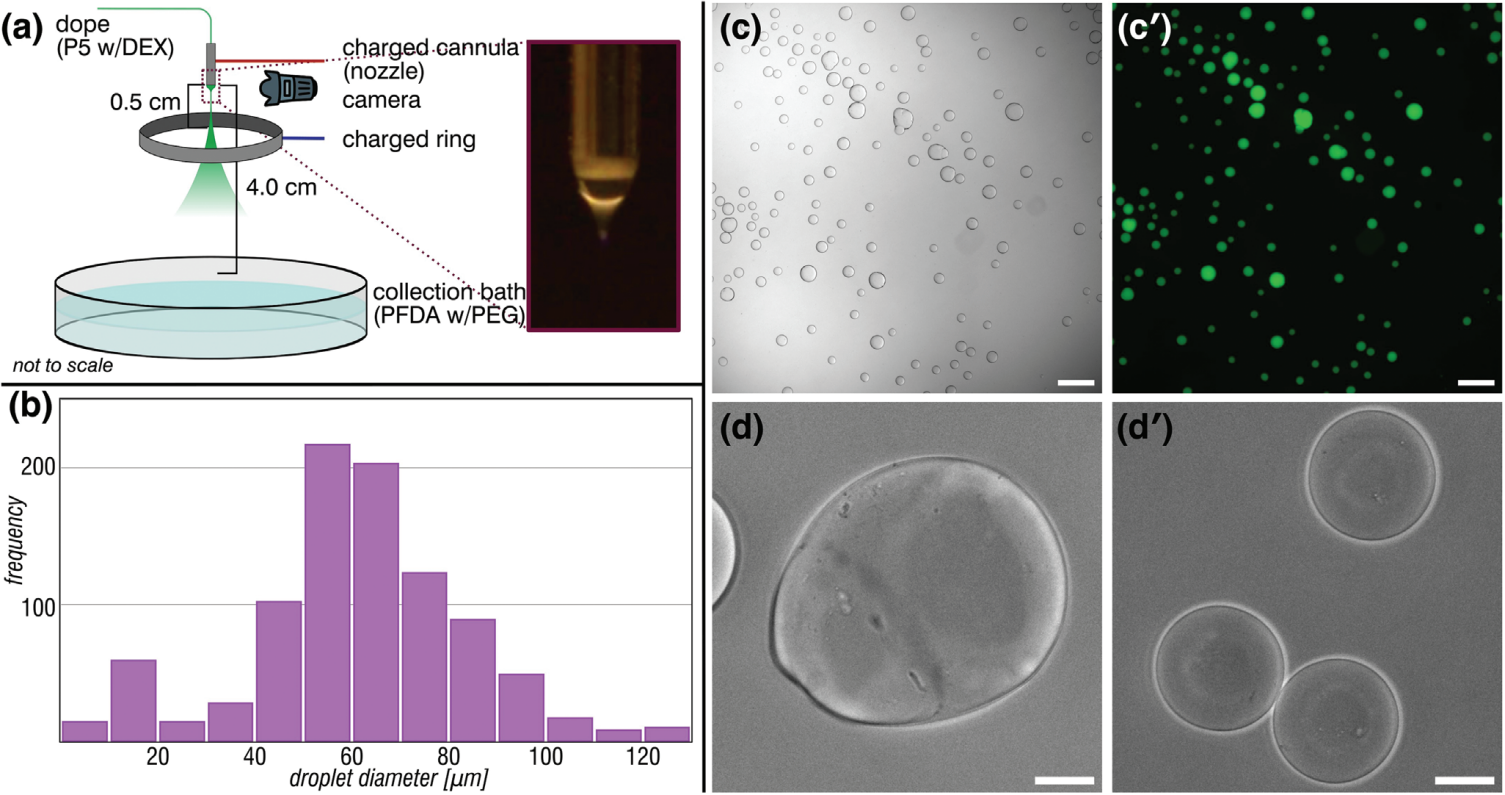
Electrospray ionization leads to efficient and controlled self‐assembly of P5–PFDA at the droplet interface. a) A schematic of the electrospray set‐up. b) Histogram of the droplet diameter distribution produced by electrospraying a P5C2M‐laden DEX phase into a PFDA‐loaded PEG bath (*n > *900). A peak is seen at a diameter of ≈65 µm, with the majority of droplets within the interval 50–80 µm. A small peak at 10–20 µm comes from satellite droplets generated during the jet breaking. c) Bright‐field images show that these electrosprayed droplets have a relatively uniform size as well as characteristic wrinkling of the droplet, aspherical shape, and non‐coalescence that indicate the formation of a film at the droplet–bath boundary. cʹ) Corresponding epifluorescence images of FITC‐dextran show that the dextran remains sequestered within the formed droplets without coalescence, indicating the presence of a boundary that prevents seepage of dextran. d) and dʹ) Zoomed‐in images of the droplets show two characteristics that a solid‐like interface has formed: elongated droplets where the fusion between two smaller droplets became arrested by the formation of the films (d), and two droplets in contact not merging due to the presence of a film surrounding them (dʹ). Scale bar in (c) and (cʹ) 500 µm; in (d) and (dʹ) 50 µm.

We electrosprayed a P5‐laden dextran solution into a bath of PFDA‐laden PEG solution. Not only could we produce DEX‐rich microdroplets at much higher throughput, but they also had a significantly more uniform size distribution (CV = 53%, 62.8 ± 1.1 µm, mean ± standard error, *n > *900) compared to glass capillary production. More importantly, we observed similar P5–PFDA assembly at the droplet interface, judged by the non‐coalescence of the formed vesicles and clear sequestration of FITC‐DEX inside. We also observed some proportion of semi‐fused droplets (**Figure** **6**d): this likely happened when the electrospray droplets impacted the PFDA‐laden PEG solution at the same location and thus started to merge. However, the rapid P5–PFDA interfacial assembly immediately prevented the complete fusion but instead resulted in an arrested coalescence. The rapid interfacial assembly can clearly be seen in these arrested structures and further proves the instantaneous nature of these interactions.

## 3 Conclusion

In this work, we have systematically characterized and manipulated the self‐assembly of fluorine‐free organic macrocycles (DAF‐P5) and perfluorinated molecules (PFAS), specifically perfluorinated diacids (PFDA). We observed that these two classes of small molecules displayed unique self‐assembly behavior, as they rapidly solidified on aqueous interfaces, creating solid, stable scaffolds templated by the contact area between aqueous solutions. Through systematic investigation and probing of the self‐assembly process, we found that DAF‐P5 and PFDA molecules self‐assemble in a manner driven by fluorophilic phase separation: the PFDA molecules gather on positively charged functional groups of the pillararene pendants, attracting more PFDA molecules and forming a self‐stabilizing phase. To the best of our knowledge, this is the first example of self‐assembly between small perfluorinated and non‐perfluorinated molecules driven by fluorophilic phase separation at either the macro‐ or microscale. Such deepened insights into the interaction of PFAS with other materials are urgently needed given the rising environmental problems associated with PFAS and the need for sequestering them.

We were additionally able to create scaffolds of DAF‐P5 and PFDA complexes in a controlled manner by utilizing ATPS, both in microfluidic devices and with electrospray. We observed that a degree of flow or mixing of the PFDA phase over the DAF‐P5 droplets is necessary to create stable scaffolds. The DAFP5‐laden aqueous droplets were able to sequester PFDA from the surrounding solution to produce stable vesicles, an approach that could be potentially useful for removing PFAS molecules from the natural aqueous environments. Furthermore, since perfluorinated molecules are fully synthetic and fluorophilic phase separation is scarcely found in supramolecular materials elsewhere, this strategy of creating self‐assembled materials is entirely orthogonal to the current toolbox available for supramolecular self‐assembly. This unique phenomenon paves the way for the construction of fluorinated scaffolds with added complexity and inspires new methods of rationally designing building blocks for supramolecular fluorophilic self‐assembly.

## 4 Experimental Section

### Materials

All chemicals for the synthesis of the pillar‐[5]‐arenes (see Scheme S1 and Figures S1‐S4, Supporting Information) were sourced from Sigma‐Aldrich. Perfluorinated diacids (PFDA‐6, PFDA‐8, PFDA‐10, and PFDA‐12) were purchased from ABCR. Poly(ethylene glycol) (PEG, *M_w_* ≈20 and ≈35 kDa), dextran from *Leuconostoc supp*. (DEX, *M_w_* ≈100, ≈150, and ≈550 kDa), and fluorescently labeled dextran (FITC‐DEX, *M_w_* ≈70 kDa) were sourced from Merck/Sigma‐Aldrich. Dulbecco's Modified Eagle Medium (DMEM, Gibco, Thermo Fischer), with 10% w/w fetal bovine serum (FBS, Gibco, Thermo Fischer), was graciously provided to us by the Cell Biology and Immunology group of Wageningen University. All chemicals were used without any further purification unless noted.

### Microfluidics

Glass capillary microfluidics devices were prepared according to procedures described by Utada et al.^[^ ^21^ ^]^ and Jampani et al. for the production of double emulsions.^[^ ^16b,c^ ^]^ In brief, cylindrical borosilicate glass capillaries (OD 0.7 mm, ID 0.5 mm, CM Scientific Ltd.) were pulled using a Narishige micropipette puller to orifice diameters ≈50 µm. A number of these capillaries and some square outer capillaries (1D 1.0 mm, Vitrotubes, CM Scientific Ltd.) were hydrophobized by plasma cleaning for 60 s and immersion in a solution of 1% v/v 1,2‐dimethyloctadecyl‐3‐aminopropylsilane (DMOAP, 42% in methanol, Sigma Aldrich) in water for 15 min with gentle shaking before being thoroughly rinsed with MilliQ water (resistivity 18.2 MΩ cm) and dried in an oven at 105 °C overnight. Devices were assembled on glass microscopy slides under a stereomicroscope, using three 21‐gauge blunt‐tip cannulas (Sterican) with notches cut in their bases as injection ports and capillaries and cannulas were fixed into place using a two‐part epoxy (Pattex Super Mix Metal). Devices were left to stand overnight to allow the glues to dry and tested for leaks before use.

Aqueous solutions were flowed through the device using an Elveflow microfluidic pressure pump (max. pressure 2 bar) and the process was imaged with a Nikon Ti2 Eclipse inverted fluorescence microscope equipped with a pE‐300ultra illumination system and a Prime BSI Express sCMOS camera. Droplets were typically obtained with applied pressures of 50 mbar in the outer PEG phase (corresponding to an approximate flow rate of *Q* ≈1 mL h^−1^) and pressures of 30 mbar in the inner DEX phase (*Q* ≈ 0.3 mL h^−1^), while pulses of ±5–25 mbar were applied in the inner phase to stimulate jet breaking.

### Electrospray

A home‐built electrospray set‐up (Figure S12, Supporting Information) was designed according to Song et al.^[^ ^20c^ ^]^ and adapted from electrospinning set‐ups used by Vats et al.^[^ ^19b^ ^]^ and Schelski et al.^[^ ^22^ ^]^ We directly used a 21‐gauge needle (inner diameter 0.51 ± 0.02 mm) as the nozzle and positive electrode, flowing the DEX solution with a pressure controller at 50 mbar applied pressure, corresponding to a flow rate of 0.39 mL h^−1^ (Figure S9, Supporting Information), and used a potential difference of 3.7 kV DC to break the jet. A stainless steel ring (diameter 5 cm, height 1 cm) served as the negative electrode, with positive and negative electrodes separated by 0.5 cm. We flowed P5C2M‐laden (0.25 mm) dextran solution (14% w/w, 100 kDa) with an Elveflow microfluidic pressure pump through the cannula while high voltage was applied. The Taylor cone was imaged with a ThorLabs Zelux 1.6 MP Color CMOS Camera with a Navitar zoom lens. Droplets were collected in a polystyrene cell culture dish (Greiner GmbH) filled with PFDA‐12‐laden (2 mm) PEG solution (9% w/w, 35 kDa), maintained at a distance of 4 cm from the needle, and were imaged using a Zeiss Axio Observer inverted optical microscope equipped with a Colibri light source and a Prime BSI Express camera.

## Conflict of Interest

The authors declare no conflict of interest.

## Author Contributions

K.R.G., T.V.M.B., H.Z., F.M.M., and S.D. performed conceptualization. K.R.G., T.N.G., L.W.H., T.V.M.B., P.V., H.Z., F.M.M., and S.D. performed methodology and validation. K.R.G., T.N.G., L.W.H., T.V.M.B., P.V., G.K.K., T.S., and F.M.M. perfromed formal analysis. K.R.G., T.N.G., L.W.H, T.V.M.B., P.V., G.K.K., and T.S. performed investigation and data curation. L.W.H., K.R.G., T.N.G., and P.V. wrote the original draft. L.W.H, K.R.G., T.N.G., T.V.M.B., P.V., T.S., G.K.K., H.Z., F.M.M., and S.D. wrote, reviewed, and edited. K.R.G., L.W.H., T.N.G., T.V.M.B., P.V., T.S., F.M.M., and S.D. performed visualization. L.W.H., T.N.G., H.Z., F.M.M., S.D. performed supervision. F.M.M. and S.D. performed project administration. L.W.H., H.Z., F.M.M., and S.D. performed funding acquisition. All authors have read and agreed to the published version of the manuscript.

## Supporting information

Supporting Information

Supplemental Video 1

Supplemental Video 2

## Data Availability

The data that support the findings of this study are available in the supplementary material of this article.
