# Supplementary material for: 2D and 3D Self‐Assembly of Fluorine‐Free Pillar‐[5]‐Arenes and Perfluorinated Diacids at All‐Aqueous Interfaces
Source: Adv Sci (Weinh). 2024 May 24;11(29):2401807. doi: 10.1002/advs.202401807 (PMC11304270; doi:10.1002/advs.202401807)
Supplement: Supplementary file 1 — Supporting Information [file ADVS-11-2401807-s002.pdf]

ADVANCED SCIENCE
Open Access

Supporting Information

for *Adv. Sci.*, DOI 10.1002/advs.202401807

2D and 3D Self-Assembly of Fluorine-Free Pillar-[5]-Arenes and Perfluorinated Diacids at All-Aqueous Interfaces

Lawrence W. Honaker, Tu-Nan Gao, Kelsey R. de Graaf, Tessa V.M. Bogaardt, Pim Vink, Tobias Stürzer, Gabriele Kociok-Köhn, Han Zuilhof, Fedor M. Miloserdov* and Siddharth Deshpande**

Supporting Information for "Two- and Three-Dimensional Self-Assembly of Fluorine-Free Pillar-[5]-Arenes and Perfluorinated Diacids at All-Aqueous Interfaces"

Lawrence W. Honaker[1,†], Tu-Nan Gao[2,3,†], Kelsey R. de Graaf[1,2,†], Tessa V. M. Bogaardt[1], Pim Vink[1], Tobias Stürzer[4], Gabriele Kociok-Köhn[5], Han Zuilhof[2,6,7*], Fedor M. Miloserdov[2,*], and Siddharth Deshpande[1,*]

[1] *Laboratory of Physical Chemistry and Soft Matter, Wageningen University & Research, 6708 WE Wageningen, The Netherlands*

[2] *Laboratory of Organic Chemistry, Wageningen University & Research, 6708 WE Wageningen, The Netherlands*

[3] *Biobased Chemistry and Technology, Wageningen University & Research, 6708 WG Wageningen, The Netherlands*

[4] *Bruker AXS GmbH, Östliche Rheinbrückenstraße 49, 76187 Karlsruhe, Germany*

[5] *Core Research Facility, University of Bath, Claverton Down, Bath BA2 7AY, United Kingdom*

[6] *School of Pharmaceutical Science and Technology, Tianjin University, Tianjin 300072, People's Republic of China*

[7] *China–Australia Institute for Advanced Materials and Manufacturing, Jiaxing University, Jiaxing 314001, People's Republic of China*

Corresponding author e-mail: han.zuilhof@wur.nl; fedor.miloserdov; siddharth.deshpande@wur.nl

Contents

1 Synthesis and Characterization of DAF-P5 Compounds **3**
1.1 Decaaminoethoxy-pillar[5]arene (**1**) (P5C2) 3
1.2 Decaaminobutoxy-pillar[5]arene (**2**) (P5C4) 4
1.3 Decaaminohexoxy-pillar[5]arene (**3**) (P5C6) 4
1.4 Decatrimethylamineethoxy-pillar[5]arene (**4**) (P5C2M) 4

2 Characterization of the P5–PFDA Complex **7**
2.1 Single Crystal X-Ray Diffraction 7
2.2 NMR Characterization 8
2.3 Isothermal Titration Calorimetry 10

3 Microfluidics and Electrospray: Determination of Flow Rates **13**

4 Additional Data and Figures **14**
4.1 Images 14
4.2 Supporting Videos 16

5 References **17**

1 Synthesis and Characterization of DAF-P5 Compounds

Compounds **4-13** were synthesized according to previously published procedures[1, 2, 3, 4, 5] (See Scheme S1). ^{1}H NMR and ^{19}F NMR spectra were recorded on a Bruker Avance III 400 MHz spectrometer at 298 K. ^{13}C NMR and electrospray ionization–mass spectrometry data were previously reported by Hu *et al.*[6] (P5C2), Fang *et al.*[7] (P5C4), Wei *et al.*[8] (P5C6), and Montes-García *et al.*[9] (P5C2M).

Scheme S1 Synthesis of DAF-P5 compounds.

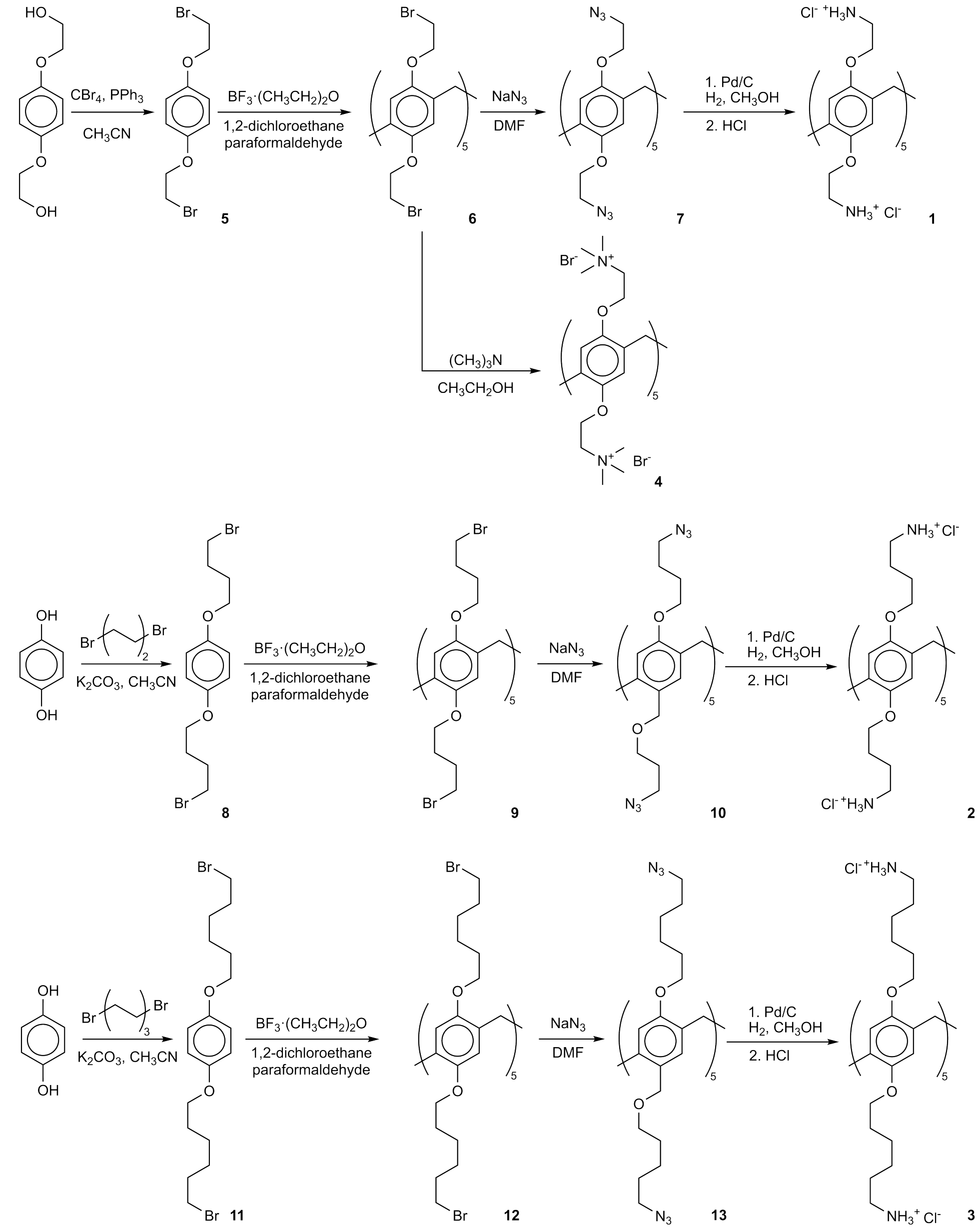

1.1 Decaaminoethoxy-pillar[5]arene (1) (P5C2)

Compound **7** (200 mg) was added to methanol (150 mL). Pd/C catalyst (160 mg) and dichloromethane (DCM) (10 mL) were added. The mixture was stirred vigorously under H_2 at room temperature for 24 h, during which the H_2 atmosphere was regenerated by vacuum refill every 3 h (there was an overnight period of 10 h without refilling H_2). After the reaction, the Pd/C was removed by filtration

and the remaining material was washed thrice with 10–15 mL methanol. Filtrate and washings were combined, treated with 1 M HCl solution in diethyl ether (6.4 mL), and concentrated under vacuum. The resultant light yellow solid (**1**) was washed with diethyl ether and dried under vacuum. Yield: 181 mg, 85%. ^{1}H-NMR of **1** (400 MHz, D_2O) δ 6.75 (s, 10H), 3.99 (t, J = 4.8 Hz, 20H), 3.91 (s, 10H), 3.25 (t, J = 4.9 Hz, 20H) (Figure S1).

1.2 Decaaminobutoxy-pillar[5]arene (2) (P5C4)

Compound **10** (200 mg) was added to methanol (150 mL). Pd/C catalyst (160 mg) and DCM (10 mL) were added. The mixture was stirred vigorously under H_2 at room temperature for 24 h, during which the H_2 atmosphere was regenerated by vacuum refill every 3 h (there was an overnight period of 10 h without refilling H_2). After the reaction, the Pd/C was removed by filtration and the remaining material was washed thrice with 10–15 mL methanol. Filtrate and washings were combined, treated with 1 M HCl solution in diethyl ether (6.4 mL), and concentrated under vacuum. The resultant light brown solid (**2**) was washed with diethyl ether and dried under vacuum. Yield: 169 mg, 80%. ^{1}H-NMR of **2** (400 MHz, Methanol-d_4) δ 6.81 (s, 10H), 3.88 (d, J = 5.2 Hz, 20H), 3.78 (s, 10H), 3.04 (t, J = 7.0 Hz, 20H), 1.90 (s, 40H) (Figure S2).

1.3 Decaaminohexoxy-pillar[5]arene (3) (P5C6)

Compound **13** (200 mg) was added to methanol (150 mL). Pd/C catalyst (160 mg) and DCM (10 mL) were added. The mixture was stirred vigorously under H_2 at room temperature for 24 h, during which the H_2 atmosphere was regenerated by vacuum refill every 3 h (there was an overnight period of 10 h without refilling H_2). After the reaction, the Pd/C was removed by filtration and the remaining material was washed thrice with 10–15 mL methanol. Filtrate and washings were combined, treated with 1 M HCl solution in diethyl ether (6.4 mL), and concentrated under vacuum. The resultant light brown solid (**3**) was washed with diethyl ether and dried under vacuum. Yield: 275 mg, 83%. ^{1}H-NMR of **3** (400 MHz, Methanol-d_4) δ 6.86 (s, 10H), 3.89 (d, J = 7.4 Hz, 20H), 3.72 (s, 10H), 2.94 (t, J = 7.7 Hz, 20H), 1.86 (d, J = 7.7 Hz, 20H), 1.72 (q, J = 8.4, 7.2 Hz, 20H), 1.62 (p, J = 7.7 Hz, 20H), 1.49 (q, J = 6.6 Hz, 20H) (Figure S3).

1.4 Decatrimethylamineethoxy-pillar[5]arene (4) (P5C2M)

This compound was prepared according to previously described procedures.[9] ^{1}H-NMR of **4** (400 MHz, D_2O) δ 7.29 (s, 10H), 4.79 (s, 20H), 4.27 (s, 10H), 4.16 – 4.11 (m, 20H), 3.56 (s, 90H) (Figure S4).

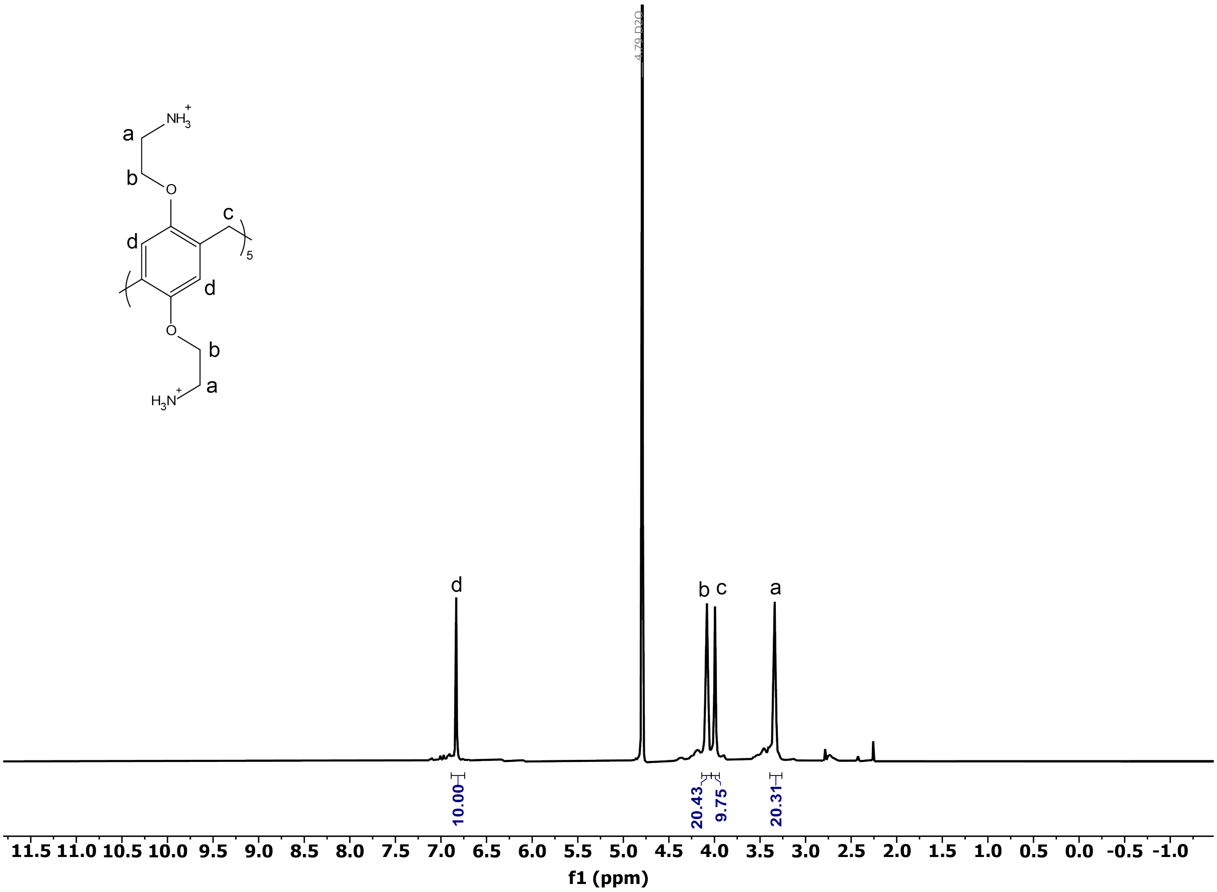

Figure S1: [1]**H NMR Spectrum of P5C2.**

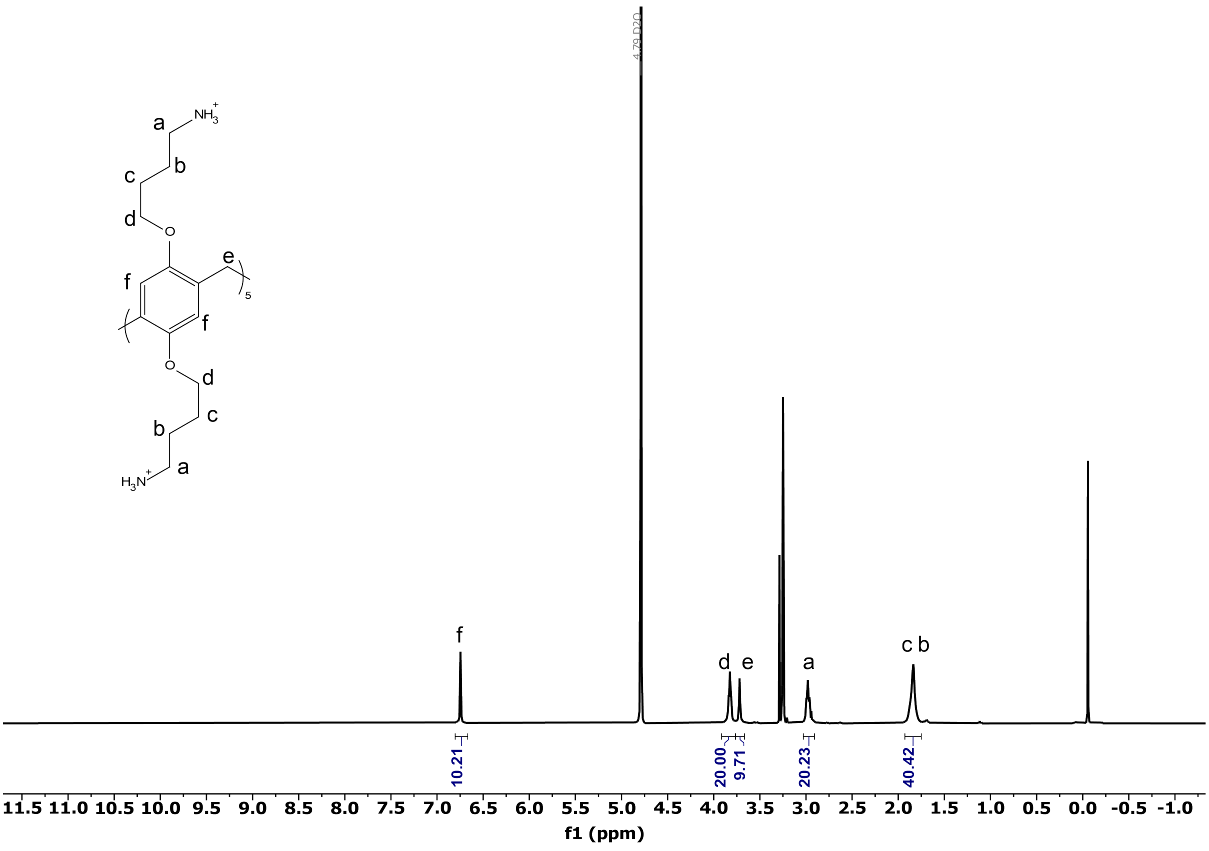

Figure S2: [1]**H NMR Spectrum of P5C4.**

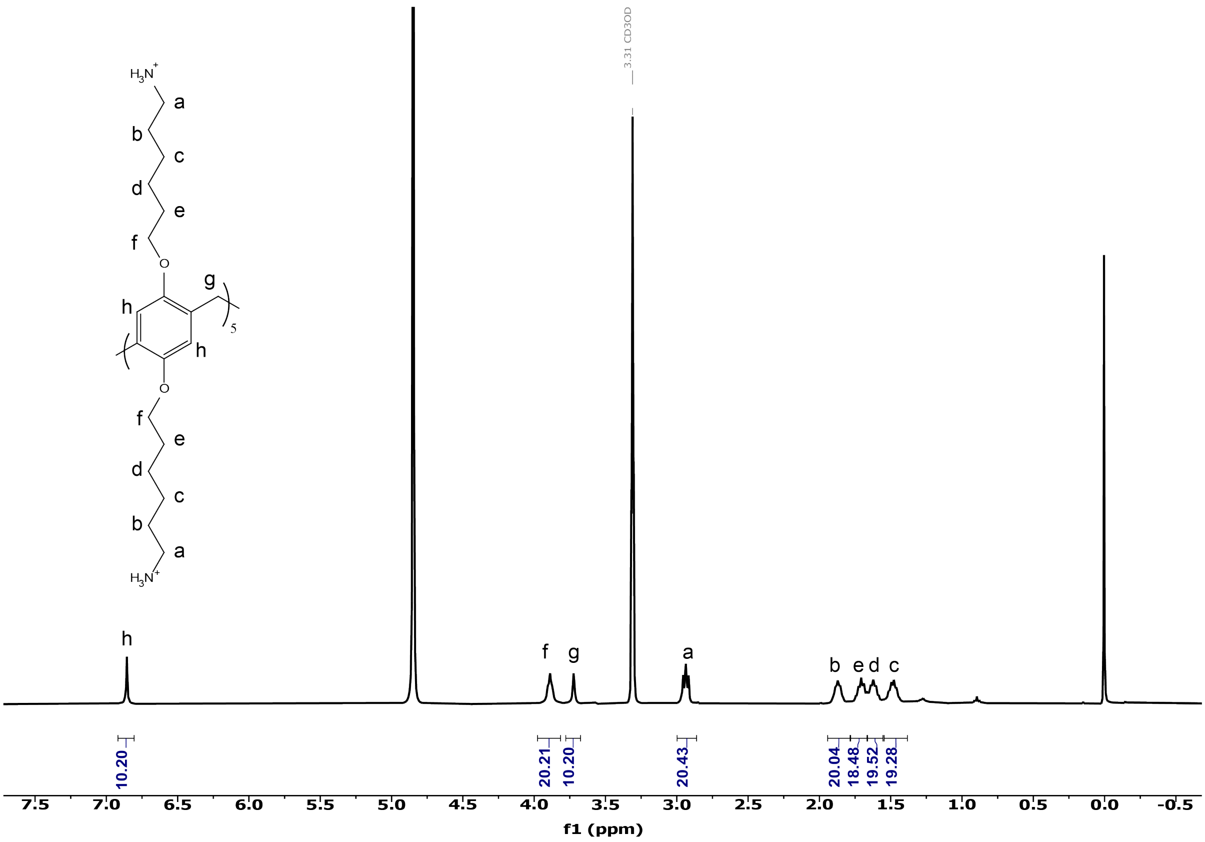

Figure S3: ^{1}**H NMR Spectrum of P5C6.**

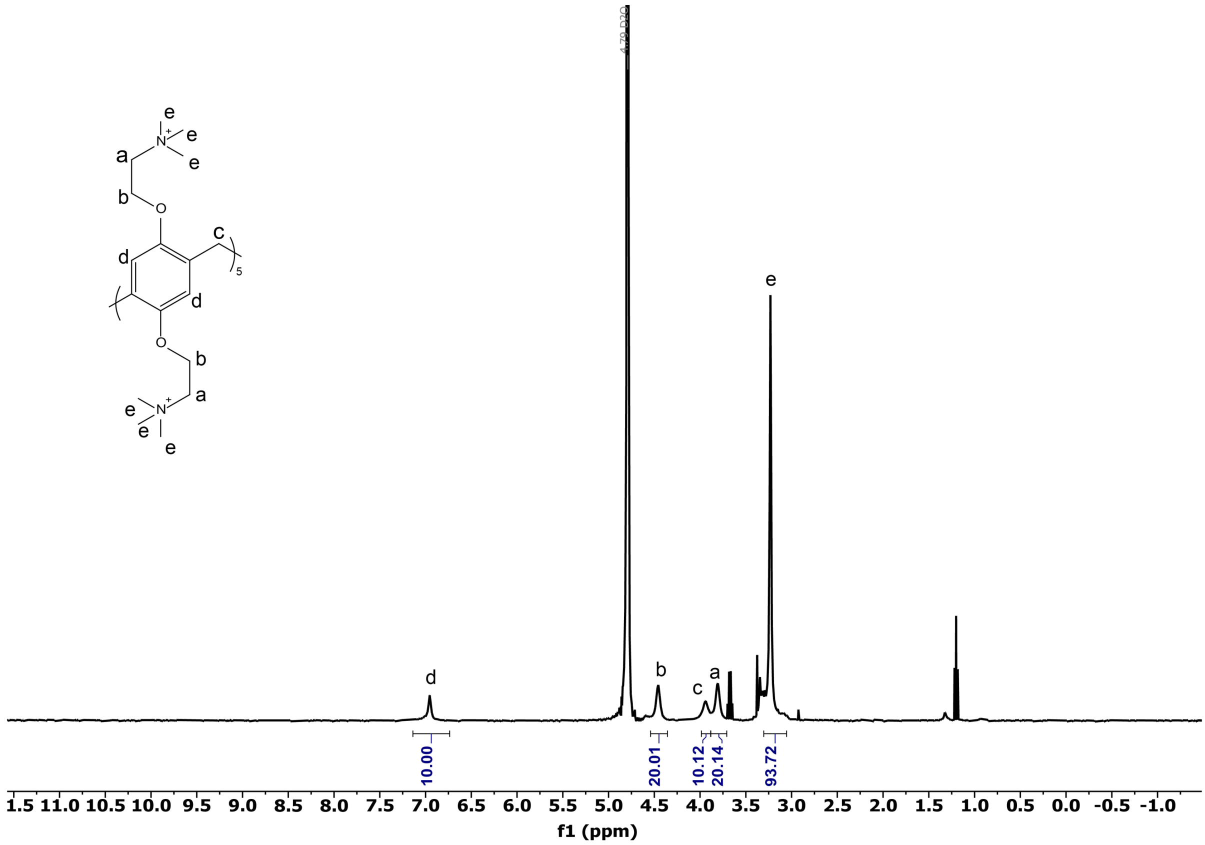

Figure S4: ^{1}**H NMR Spectrum of P5C2M.**

2 Characterization of the P5–PFDA Complex

2.1 Single Crystal X-Ray Diffraction

To prepare samples for single-crystal X-ray diffraction, we prepared aqueous solutions of 25 mg mL^{-1} PFDA-10 and 35 mg mL^{-1} P5C4. We added the P5C4 solution dropwise to the PFDA-10 solution to form a white precipitate. This precipitate was then collected and dispersed in a 1:1 mixture of acetone and water with heating to 50 °C to ensure complete dissolution of the precipitate in the solvent. This solution was then transferred to a 10 mL vial and left to sit with the cap removed to evaporate the solvent for four days, resulting in needle-like crystals which we used for X-ray diffraction measurements.

X-ray intensity data were measured on a Bruker D8 VENTURE system equipped with a Ga METALJET (Ga K_α = 1.34139 Å) with multilayer optics monochromator and a PHOTON III M28 detector. All data was processed using the Bruker Single-Crystal package APEX4 2022.1-1 with SAINT 8.40B and SADABS 2016/2. The structure was solved with SHELXT and refined by a full-matrix least-squares procedure based on F^2 (Shelxl-2019/2).

The crystal diffracted poorly and reflections beyond 0.95 Å resolution could not clearly be observed. The lack of strict long-range order and therewith missing high-resolution diffraction is expected for such a flexible structure. This manifests itself in the plentiful disorder of which some could be resolved. The pillar-[5]-arene molecule shows disorder in three of the ten C4-chains, each with 1:1 disorder. One entire PFDA-10 chain is disordered over two sites in a 60:40 ratio. All minor parts of the disorders were refined as being geometrically identical to their major part. There is potential partial disorder in the other four PFDA-10 molecules; however, it was not possible to resolve this.

The cavity of the pillar-[5]-arene contains acetone which could be modelled with the disordered structure refinement module from D. Kratzert *et al.*[11]. In total, two molecules of acetone disordered over two sites at a 1:1 ratio and with occupation of 25% per disorder. There is more solvent in the voids. However, due to a lack of high-angle resolution data, it was not possible to assign peaks from the Difference Fourier map to specific molecules and model this.

All atoms were refined with bond lengths, angle and ADP restraints and constraints. Hydrogen atoms were placed onto calculated positions and refined using a riding model. A shift limiting restraint was used in the final stages of the refinement to assist convergence. Additional programs used for analyzing data and their graphical manipulation included SHELXle5[12] and Mercury[13].

Table S1: Crystal data and structure refinement for the P5C4–PFDA-10 complex.

**Identification code**	2023s016_p5pfsa	
**Empirical formula**	$C_{128} H_{136} F_{80} N_{10} O_{31}$	
**Formula weight**	3830.46	
**Temperature**	100(2) K	
**Wavelength**	1.34139 Å	
**Crystal system**	Orthorhombic	
**Space group**	Pbca	
**Unit cell dimensions**	a = 27.2838(12) Å	$\alpha = 90°$
	b = 22.9643(11) Å	$\beta = 90°$
	c = 54.007(3) Å	$\gamma = 90°$
**Volume**	33838(3) Å^3	
**Z**	8	
**Density (calculated)**	1.504 Mg m^{-3}	
**Absorption coefficient**	0.943 mm^{-1}	
**F000**	15536	
**Crystal size**	n/a	
**Theta range for data collection**	1.423-57.691°	
**Index ranges**	$h = [-34, 34]$	
	$k = [-28, 28]$	
	$l = [-67, 67]$	
**Reflections collected**	707819	
**Independent reflections**	34716 [R(int) = 0.1066]	
**Completeness to $\theta = 53.594°$**	99.9%	
**Absorption correction**	Semi-empirical from equivalents	
**Max. and min. transmission**	0.6689 and 0.5223	
**Data / restraints / parameters**	34716 / 9870 / 2687	
**Goodness-of-fit on F^2**	3.253	
**Final *R* indices ($I > 2\sigma(I)$**	R1 = 0.3366, wR2 = 0.7205	
***R* indices (all data)**	R1 = 0.3667, wR2 = 0.73815	
**Extinction Coefficient**	n/a	
**Largest diffraction peak and hole**	1.481 and -1.273 e.Å^{-3}	

2.2 NMR Characterization

For the NMR characterization of the P5C4–PFDA-10 complex, 35 mg mL^{-1} P5C4 solution was added to an aqueous solution of 25 mg mL^{-1} PFDA-10 dropwise to form a white precipitate. This precipitate was then collected and washed with water before being dried at 100 °C for 30 min, obtaining a slightly white powder. We then added the powder to an NMR tube and dissolved the compound in 0.4 mL deuterated dimethyl sulfoxide (DMSO-d_6), with 1 µL 1-bromo-4-fluorobenzene added as an internal ^{19}F NMR standard. The NMR tube was then heated with a heat gun to ensure dissolution of the compound. A decoupled ^{19}F NMR spectrum was measured overnight with 128 scans, spaced at intervals of 30 s, while the ^{1}H NMR spectrum was collected with 64 scans and intervals of 15 s.

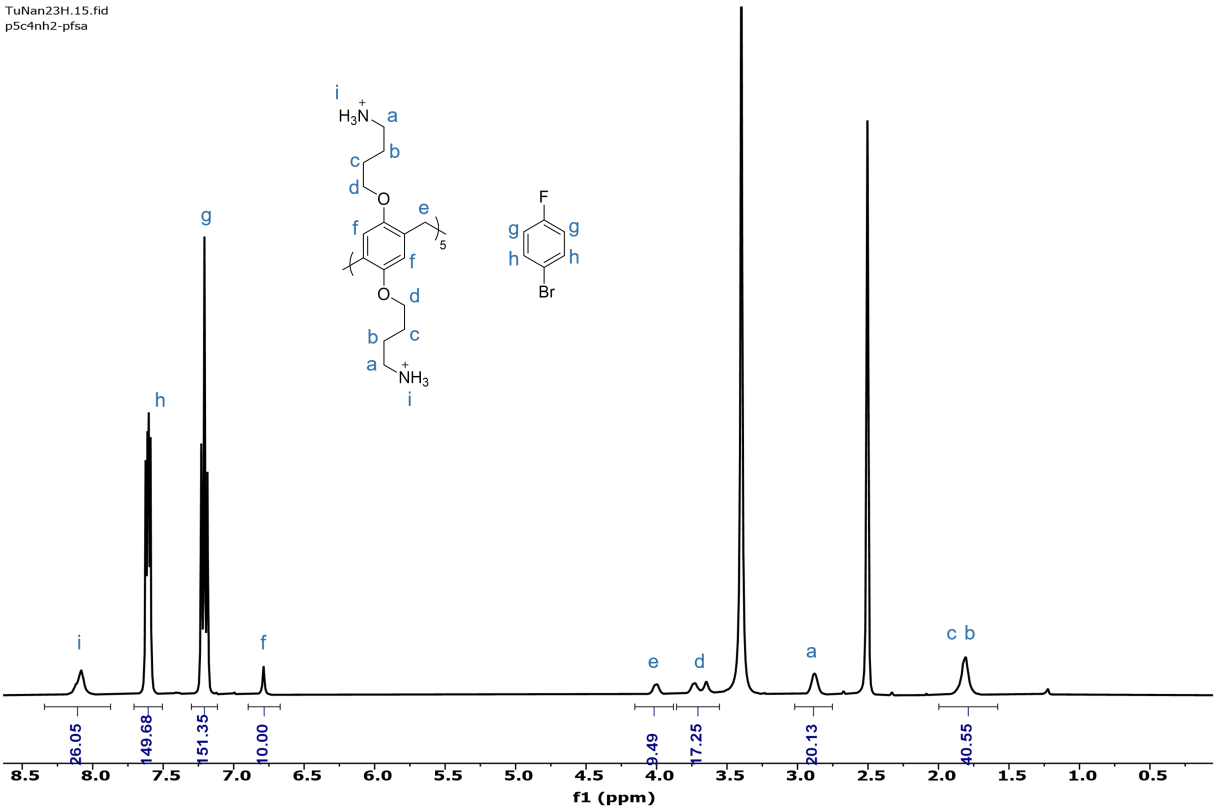

Figure S5: [1]**H NMR spectrum of the complex formed between P5C4 and PFDA-10 in DMSO-*d*$_6$.** Peaks assigned to the internal standard are indicated (g-h).

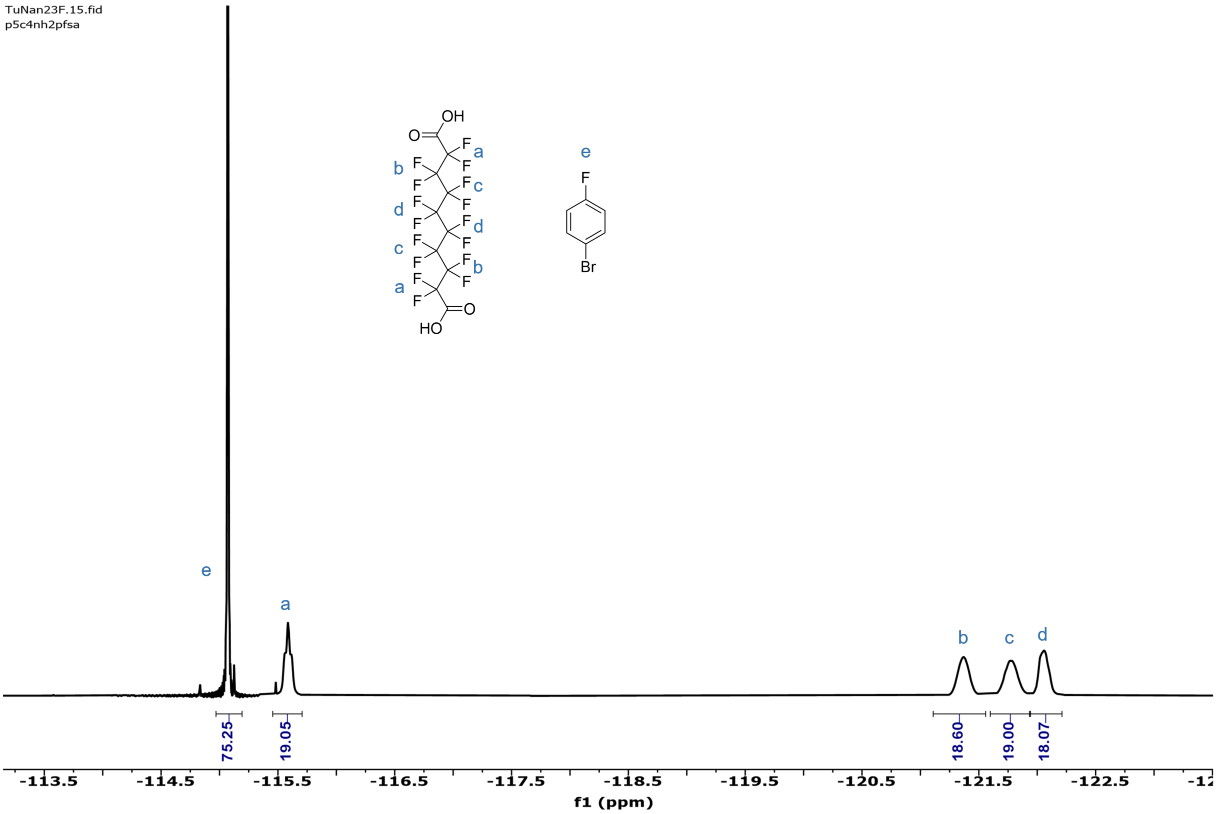

Figure S6: [19]**F NMR spectrum of the complex formed between P5C4 and PFDA-10 in DMSO-*d*$_6$.** The peak assigned to the internal standard is indicated as (e).

2.3 Isothermal Titration Calorimetry

Isothermal titration calorimetry measurements on the PFDA–P5 complexes were performed with a MicroCal VP-ITC isothermal titration calorimeter. A reverse-mode ITC protocol was used due to the poor solubility of the perfluorinated compounds which does not allow us to run a normal mode ITC and achieve saturation for 1:5 ratio binding. A 20 mM phosphate buffer with pH 7.4 was used for both the P5 and the PFDA solutions. The P5 was held in the injection syringe at a concentration of 1 mM and the PFDA was in the cell at a concentration of 0.5 mM. The titrations were performed twice with reported values the average of duplicate experiments. Results are presented in both Table S2 and in Figure S7.

Table S2: Isothermal titration calorimetry data between different P5 and PFDA molecules (from Figure S7). Top rows are the binding constants; bottom rows are the experimentally determined binding ratios. '–' in a cell indicates no conclusive measurement was obtainable. Binding constants are expressed in M^{-1}.

	**P5C2**	**P5C4**	**P5C2M**
**PFDA-8**	**–** (a)	$(3.58 \pm 0.32) \times 10^3$ (b)<br>1 : 4.0	**–** (c)
**PFDA-10**	$(9.22 \pm 2.41) \times 10^5$ (d)	$(4.52 \pm 0.30) \times 10^4$ (e)<br>1 : 4.6	$(4.46 \pm 0.35) \times 10^4$ (f)
**PFDA-12**	$(7.61 \pm 2.56) \times 10^6$ (g)<br>1 : 5.4	$(9.04 \pm 3.42) \times 10^6$ (h)<br>1 : 4.6	$(8.86 \pm 1.52) \times 10^6$ (i)<br>1 : 5.2

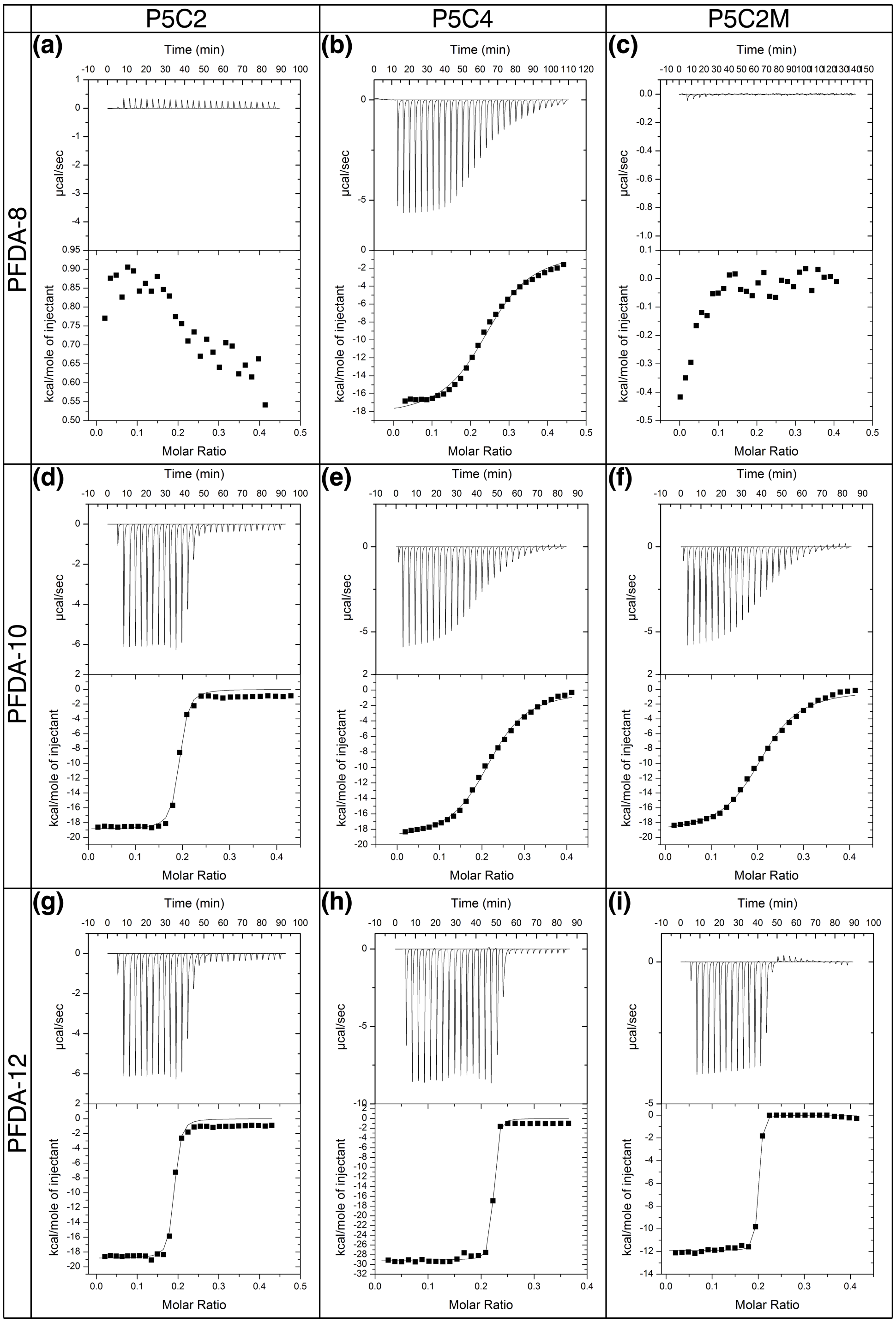

Figure S7: **Isothermal Titration Calorimetry measurements generally confirm 1:5 binding ratios for most P5–PFDA complexes.** We performed ITC measurements on the complexes of (a) P5C2–PFDA-8; (b) P5C4–PFDA-8; (c) P5C6–PFDA-8; (d) P5C2–PFDA-10; (e) P5C4–PFDA-10; (f) P5C6–PFDA-10; (g) P5C2–PFDA-12; (h) P5C4–PFDA-12; and (i) P5C6–PFDA-12. We used a constant concentration of 1 mM P5 and 0.5 mM PFDA for each measurement. With PFDA-10 and PFDA-12, we generally find a binding ratio of ∼1:5, in good agreement with theoretical predictions and the NMR data.

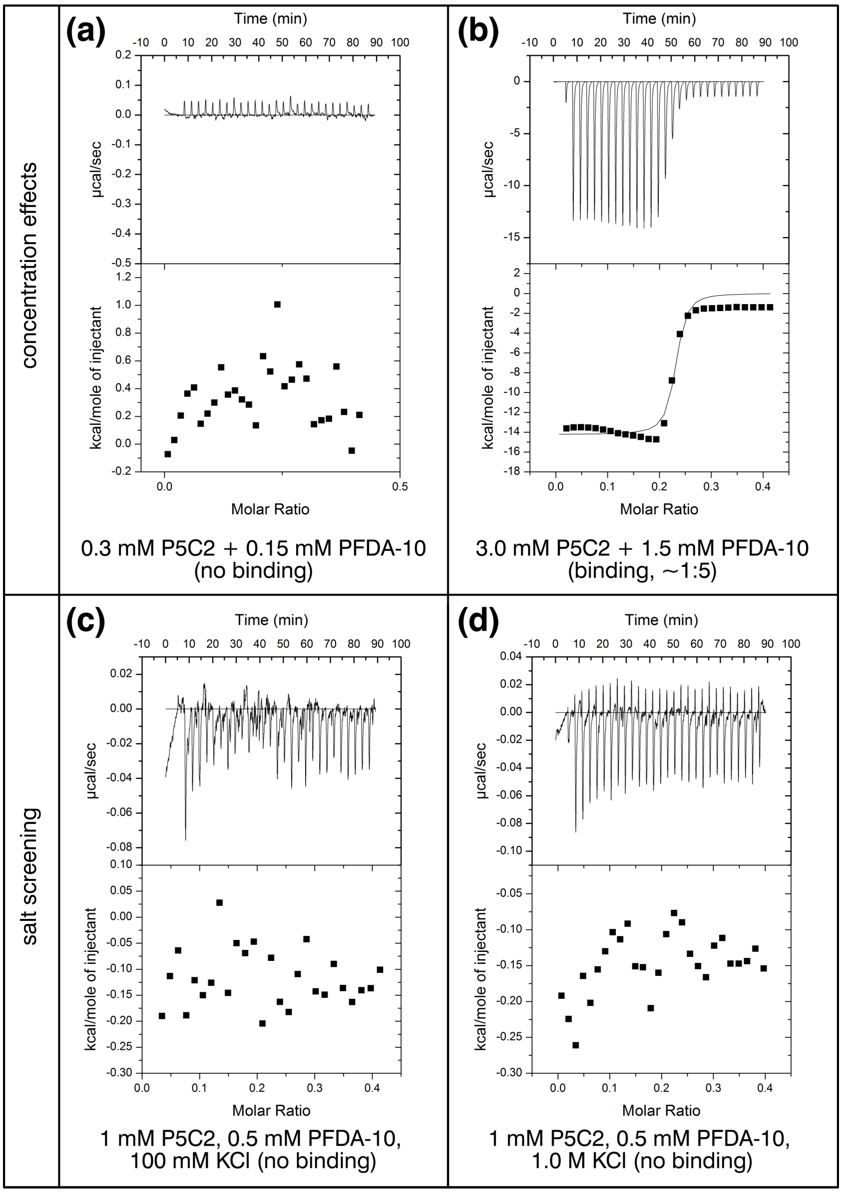

Figure S8: **Isothermal Titration Calorimetry data show that the formation of complexes can be affected by both concentrations of the species present as well as salt screening.** (a-b) We performed ITC measurements on complexes of P5C2–PFDA-10 (corresponding to Figure S7(d)), varying the concentrations of P5 and PFDA to (a) 0.3 mM P5C2 and 0.15 mM PFDA-10, which showed no binding constant, and (b) 3.0 mM P5C2 and 1.5 mM PFDA-10, which shows a binding constant comparable to that obtained with the 1.0 mM : 0.5 mM ratio measured in Figure S7(d). (c-d) We also attempted to perform ITC measurements with salt concentrations of (c) 100 mM and (d) 1 mM KCl, both of which showed a binding constant $K < 10^3$ M^{-1}.

3 Microfluidics and Electrospray: Determination of Flow Rates

We used pressure pumps (Elveflow) to flow our dextran solutions both in microfluidics and for electrospray. As these solutions are incompressible fluids, we can relate the pressure applied by the controller ΔP to the volumetric flow rate Q through a tube with the Hagen–Poiseuille equation:

$$\Delta P = \frac{8\eta L Q}{\pi R^4} \tag{S1}$$

where L is the tubing length, η the fluid viscosity, and R the tubing diameter. This estimation is presented in Figure S9.

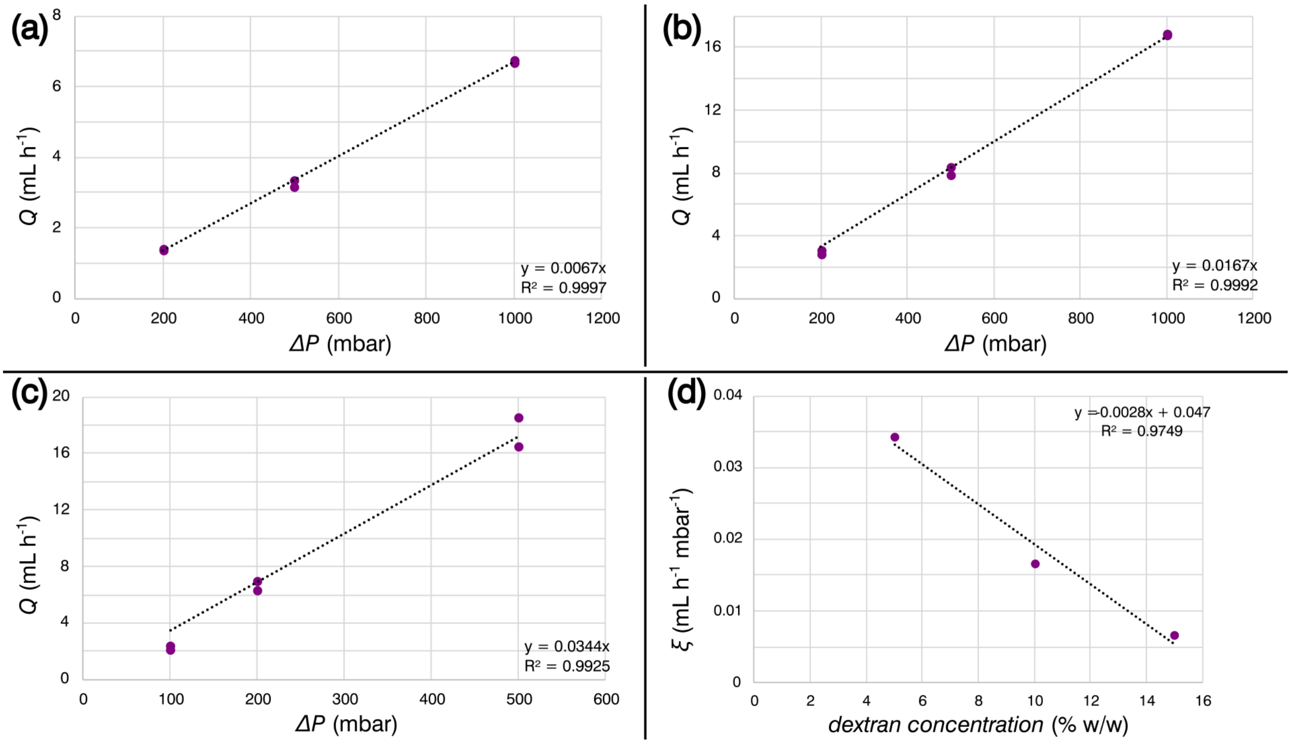

Figure S9: **The Hagen–Poiseuille equation can be used to relate the applied pressures to the flow rates of the dextran solutions during electrospray.** (a-c) Flow rates of the inner dextran phases (M_w ~100 kDa, with a-c respectively 15, 10 and 5 w/w%) are plotted against applied pressures. Applying the linear relation between ΔP and Q from the Hagen–Poiseuille equation (Equation S1), we determine the conversion factor ξ (mL h^{-1} mbar^{-1}). (d) By plotting ξ against dextran concentrations, assuming these relate linearly to fluid viscosity, we can interpolate ξ for various dextran solutions and thus estimate the experimental flow rates.

4 Additional Data and Figures

4.1 Images

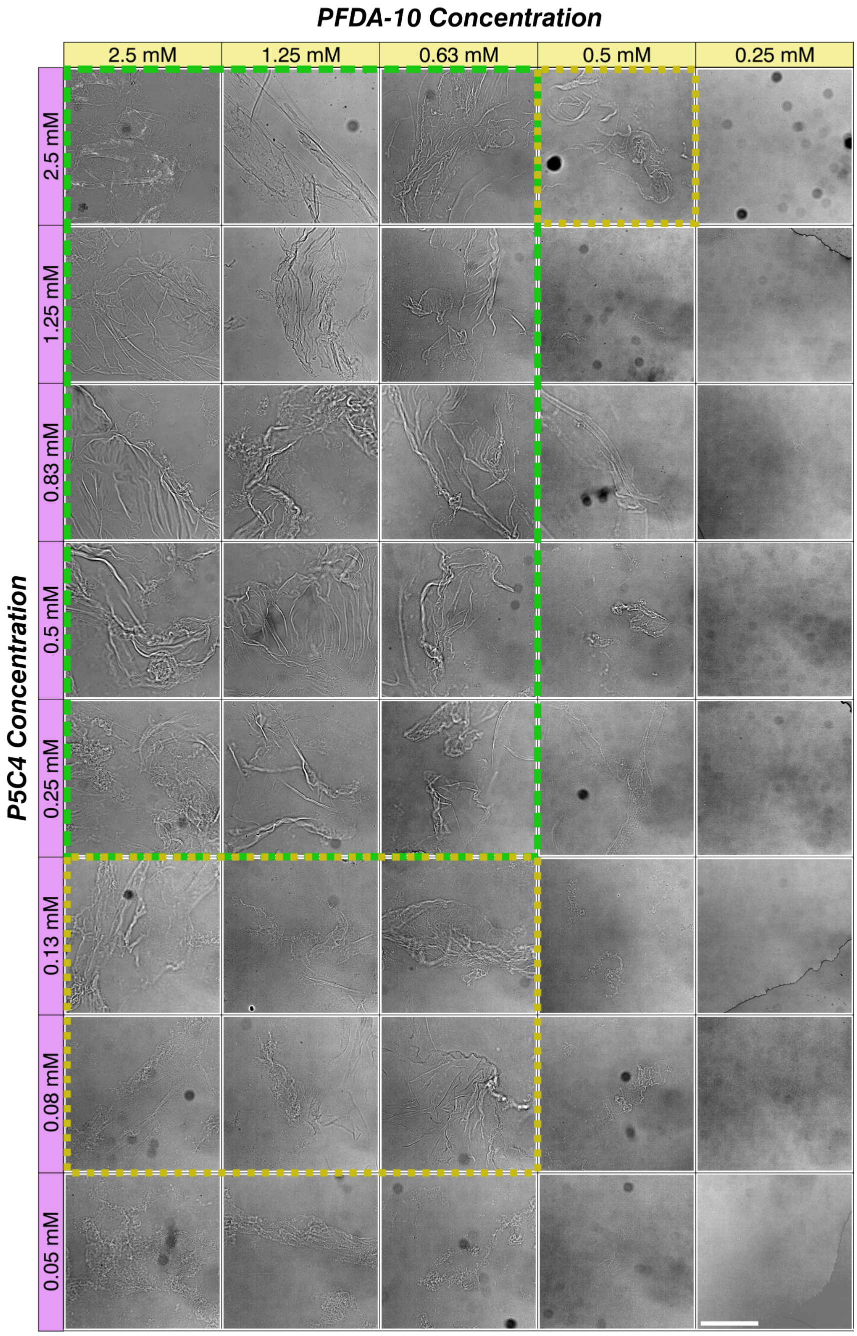

Figure S10: **The self-assembly process occurs above specific threshold concentrations of P5 and PFDA and is also dependent on their relative ratio.** In all combinations between solutions of 0.25–2.5 mM P5C4 and 0.63–2.5 mM PFDA-10, we see clear and unmistakable film formation with brightfield microscopy (green box). Some other combinations of other concentrations give hints of assembling structures (yellow boxes). For the rest of the ratios and absolute concentrations, we do not see any visible self-assembly. Scale bar 500 µm.

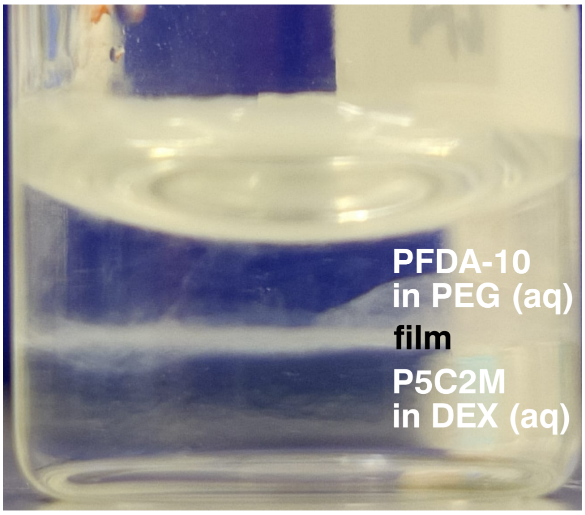

Figure S11: **The formation of a P5-PFDA film can be templated by the interface between two aqueous phases.** A PFDA-10-containing aqueous PEG phase (17% w/w, M_w 20 kDa) floated on top of a P5C2M-laden aqueous dextran solution (10% w/w, M_w 100 kDa) gives rise to a macroscopically visible film that is shaped by the phase boundary. The ultralow interfacial tension between the two aqueous solutions allows for a nearly flat interface.

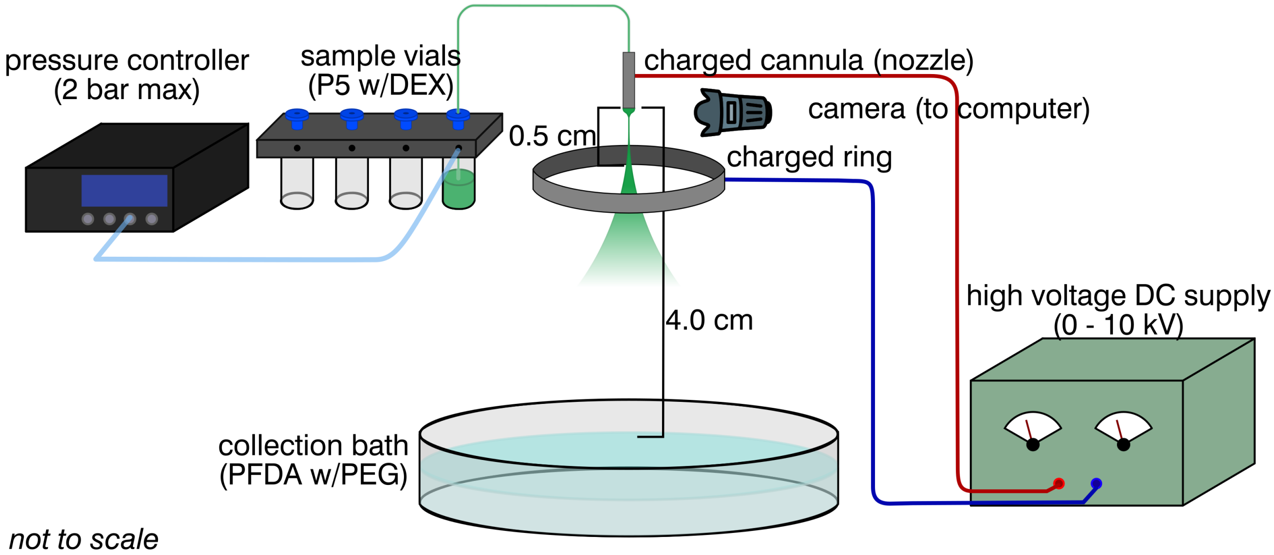

Figure S12: **Schematic of the electrospray set-up used.** A charged ring is used as the anode, while the cannula through which the dextran-P5 solution flows is used as the cathode. A constant distance between cathode and anode as well as the ring and the collection bath is maintained. Voltage is supplied with a high voltage DC supply. The dextran-P5 solution is pumped through the cannula using a pressure pump. The spray is collected into a PEG solution laden with PFDA.

4.2 Supporting Videos

SI Video 1 *The formation of the complex between a PFDA and P5 occurs rapidly, visible both to the unaided eye and at the microscopic level.*

SI Video 2 *Complexes of combinations of PFDA-10 with different P5 molecules (P5C2, P5C4, and P5C6) show different degrees of resistance to rupture and breaking, indicating some effects of size matching.*

5 References

[1] T. Ogoshi, S. Kanai, S. Fujinami, T.A. Yamagishi, Y. Nakamoto, *para*-Bridged Symmetrical Pillar[5]arenes: Their Lewis Acid Catalyzed Synthesis and Host–Guest Property. Journal of the American Chemical Society **130**, 5022–5023 (2008). `https://doi.org/10.1021/ja711260m`

[2] T. Ogoshi, M. Hashizume, T.A. Yamagishi, Y. Nakamoto, Synthesis, conformational and host–guest properties of water-soluble pillar[5]arene. Chemical Communications **46**, 3708–3710 (2010). `https://doi.org/10.1039/c0cc00348d`

[3] T. Ogoshi. Synthesis of novel pillar-shaped cavitands "pillar[5]arenes" and their application for supramolecular materials. Journal of Inclusion Phenomena and Macrocyclic Chemistry **72**, 247–262 (2012). `https://doi.org/10.1007/s10847-011-0027-2`

[4] W. Xue, P.Y. Zavalij, L. Isaacs, Pillar[n]MaxQ: A New High Affinity Host Family for Sequestration in Water. Angewandte Chemie - International Edition **59**, 13313–13319 (2020). `https://doi.org/10.1002/anie.202005902`

[5] N. Song, T. Kakuta, T. Yamagishi, Y.W. Yang, T. Ogoshi, Molecular-Scale Porous Materials Based on Pillar[n]arenes. Chem **4**, 2029–2053 (2018). `https://doi.org/10.1016/J.CHEMPR.2018.05.015`

[6] X.-B. Hu, L. Chen, W. Si, Y. Yu, J.-L. Hou, Pillar[5]arene decaamine: synthesis, encapsulation of very long linear diacids and formation of ion pair-stopped [2]rotaxanes. Chemical Communications **47**(16), 4694–4696 (2011). `https://doi.org/10.1039/C1CC10633C`

[7] Y. Fang, X. Yuan, L. Wu, Z. Peng, W. Feng, N. Liu, D. Xu, S. Li, A. Sengupta, P.K. Mohapatra, L. Yuan, Ditopic CMPO-pillar[5]arenes as unique receptors for efficient separation of americium(III) and europium(III). Chemical Communications **51**(20), 4263–4266 (2015). `https://doi.org/10.1039/C4CC09248A`

[8] L. Wei, Y. Fang, Y. Jia, Y. Yang, J. Liao, N. Liu, X. Yang, W. Feng, J. Ming, L. Yuan, Pillar[5]arene-based diglycolamides for highly efficient separation of americium(III) and europium(III). Dalton Transactions **43**(10), 3835–3838 (2014). `https://doi.org/10.1039/C3DT53336K`

[9] V. Montes-García, C. Fernández-López, B. Gómez, I. Pérez-Juste, L. García-Río, L.M. Liz-Marzán, J. Pérez-Juste, I. Pastoriza-Santos, Pillar[5]arene-Mediated Synthesis of Gold Nanoparticles: Size Control and Sensing Capabilities. Chemistry – A European Journal **20**(27), 8404–8409 (2014). `https://doi.org/10.1002/chem.201402073`

[10] T.N. Gao, S. Huang, R. Nooijen, Y. Zhu, G. Kociok-Köhn, T. Stürzer, G. Li, G. Salentijn, B. Chen, H. Bitter, F.M. Miloserdov, H. Zuilhof, Rim-Based Binding of Perfluorinated Acids to Pillararenes Purifies Water. Angewandte Chemie – International Edition (2024). `https://doi.org/10.1002/anie.202403474`.

[11] D. Kratzert, I. Krossing, Recent improvements in *DSR*. Journal of Applied Crystallography **51**(3), 928–934 (2018). `https://doi.org/10.1107/S1600576718004508`.

[12] C.B. Hübschle, G.M. Sheldrick, B. Dittrich, *ShelXle*: a Qt graphical user interface for *SHELXL*. Journal of Applied Crystallography **44**(6), 1281–1284 (2011). `https://doi.org/10.1107/S0021889811043202`.

[13] C.F. Macrae, P.R. Edgington, P. McCabe, E. Pidcock, G.P. Shields, R. Taylor, M. Towler, J. Streek, *Mercury*: visualization and analysis of crystal structures. Journal of Applied Crystallography **39**(3), 453–457 (2006) `https://doi.org/10.1107/S002188980600731X`
